# The poplar pathogen Sphaerulina musiva has a dynamic genome architecture marked by chromosomal inversions and changes in transposable element abundance

**DOI:** 10.1099/mgen.0.001603

**Published:** 2026-01-07

**Authors:** Alex Z. Zaccaron, Alexandre Lassagne, Kelsey L. Søndreli, Martha A. Sudermann, Ricardo I. Alcalá Briseño, Niklaus J. Grünwald, Alexandra J. Weisberg, Jared M. LeBoldus

**Affiliations:** 1 Department of Botany and Plant Pathology, Oregon State University, Corvallis, OR, USA; 2 Horticultural Crops Disease and Pest Management Research Unit, USDA Agricultural Research Service, Corvallis, OR, USA; 3 Department of Forest Engineering, Resources, and Management, Oregon State University, Corvallis, OR, USA

**Keywords:** effectors, mesosynteny, pangenome, repeat-induced point mutations, transposons, two-speed genome

## Abstract

Fungal plant pathogens possess dynamic genomes, frequently shaped by transposable elements, that enable rapid adaptation to adverse conditions and host resistance mechanisms. However, assessing the adaptive significance of these genomic features remains challenging, in part due to the lack of high-quality genome assemblies for multiple members of a given species. To gain insights into genomic factors shaping pathogen evolution, we sequenced and assembled near-chromosome-scale genomes of 18 geographically diverse North American isolates of *Sphaerulina musiva*, a significant, important pathogen causing Septoria leaf spot and stem canker disease of poplar trees. Comparative genomic analyses indicated that all isolates possess 13 chromosomes with no evidence of accessory chromosomes. Transposable element (TE) content varied considerably among isolates (6.8 %–15.7 %), with a higher abundance in isolates from Oregon, British Columbia and Alberta, geographic regions outside the native range of *S. musiva*. The variation in TE content largely explained differences in genome size among isolates and suggested lineage-specific proliferation of TEs. Although a gene-based pangenome analysis indicated a relatively low percentage (9.5%) of accessory genes, this subset was enriched for candidate effectors. Our results indicate that *S. musiva* exhibits features of a ‘one-speed genome’ model. However, increased TE content is correlated with longer intergenic regions of candidate effector genes, suggesting that proliferation of TEs may be driving increased compartmentalization. Finally, synteny analysis revealed a total of 43 long chromosomal inversions with an average size of 293 kb that covered 34% of the *S. musiva* genome. These chromosomal inversions were more frequently observed in isolates from the pathogen’s native range in the Eastern USA, and at least one inversion was predicted to affect the organization of a secondary metabolite gene cluster. These findings provide novel insights into the genome structure, TE dynamics and chromosomal rearrangements of the poplar pathogen *S. musiva*, offering a foundation for understanding its evolution and adaptation across diverse geographic regions and host species.

**Impact Statement**

Poplar trees are a valuable source of fibre and other wood-based products. However, they are often susceptible to *Sphaerulina musiva*, a fungal pathogen causing Septoria leaf spot and stem canker disease. The domestication and expansion of poplar cultivation facilitated the movement of *S. musiva* to western North America, giving rise to new lineages of the pathogen that co-evolve with native hosts. While previous population genomic studies have investigated the genetic diversity of *S. musiva* lineages, changes in genome organization and structural variation remained unexplored. Prior to this study, only a single reference genome was available for *S. musiva*, limiting our understanding of its genomic diversity and structure. Our analysis of 18 near-chromosome-scale genomes mitigates these limitations, providing novel insights into the genome organization of *S. musiva*. Notably, one of these genome assemblies, that of isolate MN-14, is gapless without unplaced contigs, which represents a new high-quality reference genome for *S. musiva*. The structural diversity includes extensive chromosomal inversions and lineage-specific transposable element proliferation. These findings greatly expand our understanding of fungal plant pathogen evolution.

## Data Summary

The sequences generated in this study are available in the National Center for Biotechnology Information (NCBI) under BioProject accession PRJNA1198341. Sequenced Nanopore and Illumina reads have been deposited at the NCBI Sequence Read Archive (SRA). A complete list of SRA accession numbers is available in Table S1 (available in the online Supplementary Material). Scripts and code used in this study are available in a public GitHub repository available at https://github.com/alexzaccaron/2025_smusiva_chr.

## Data Availability

All supporting data, code and protocols have been provided within the article or through supplementary data files. Sixteen supplementary tables and nine supplementary figures are available with the online version of this article.

## Introduction

Understanding the genetic diversity of fungal pathogens is crucial for developing sustainable disease management strategies. Many filamentous fungal plant pathogens exhibit remarkable genome plasticity, which enables them to rapidly adapt to adverse conditions and different hosts [1]. This plasticity originates from various genomic factors that can greatly vary within or between species, such as genome size, chromosome number, chromosomal rearrangements and other structural variations [2,5]. These dynamic genomic elements collectively enable fungal pathogens to evolve quickly, posing significant challenges to plant protection efforts across various ecosystems.

A key feature typically associated with genome plasticity is the peculiar architecture of fungal pathogen genomes, often described as the ‘two-speed genome’ model [6,8]. This model is characterized by a bipartite genome architecture composed of regions rich in repetitive DNA interspersed with regions poor in repetitive DNA. Because genes important for pathogenicity are frequently located in repeat-rich regions, this genome organization is expected to promote adaptive genomic changes through point mutations [9], insertions [10] and deletions [11] induced by the presence or mobility of TEs. As a result, these repeat-rich regions are believed to evolve at a faster pace compared to repeat-poor regions, potentially explaining the rapid adaptability of fungal pathogens. However, while the ‘two-speed genome’ concept has been proposed for many fungal pathogens [7,12], the extent to which lineage-specific expansion of TEs affects genome compartmentalization remains poorly explored.

The presence/absence patterns of genes important for pathogenicity can vary between closely related lineages of fungal pathogens [13,15]. This variability presents a challenge when using single reference genomes for comprehensive analyses of genetic diversity within pathogen populations. To address this limitation, researchers have increasingly adopted pangenome analyses of populations [16,18]. The pangenome encompasses two main components: the ‘core’ genome, which refers to genes present in all isolates, and the accessory genome, which refers to genes in some but not all isolates [19]. Pangenome analyses have become the current standard for population genomic studies of fungal pathogens, largely due to the affordability of long-read sequencing technologies.

*Sphaerulina musiva* (Peck) Quaedvlieg, Verkley and Crous (syn. *Septoria musiva* Peck; teleomorph=*Mycosphaerella populorum* Thompson) is a fungal pathogen member of the class Dothideomycetes (Capnodiales; Mycosphaerellaceae) and the cause of Septoria leaf spot and stem canker disease in poplar trees (*Populus* spp.) [20,21]. *S. musiva* initiates colonization through natural openings and wounded tissues of the host [22]. After a period of biotrophic growth, the pathogen transitions to necrotrophy, causing leaf spots, progressing to defoliation, reduced photosynthetic capacity and formation of stem cankers that increase the risk of stem breakage and tree mortality [20,21]. *S. musiva* is endemic to the eastern USA, which encompasses the native range of its ancestral host, *Populus deltoides* [23,24]. However, the pathogen has been introduced to other regions of the Americas, including Argentina [25], Brazil [26], Canada [27] and the Pacific Northwest [24,28]. In Canada and the Pacific Northwest, *S. musiva* is commonly associated with non-native hosts, particularly *Populus trichocarpa* and *Populus balsamifera*, indicating that the spread of *S. musiva* was accompanied by host shifts [21,24, 27]

The first reference genome for *S. musiva* was obtained for isolate SO2202 from Quebec, Canada [29,30]. The genome sequencing was conducted at the Joint Genome Institute (JGI) using a combination of Roche 454 and Illumina Solexa platforms. Genome assembly efforts resulted in 13 scaffolds with a total size of 29 Mb, representing the 13 chromosomes of *S. musiva*. This reference genome of isolate SO2202 has proven highly useful for subsequent population studies investigating the genetic diversity of *S. musiva* [28,31]. It has also been used in functional studies aimed at developing strategies to manage Septoria leaf spot and stem canker [32,34]. However, chromosome-scale differences and structural variation within *S. musiva* isolates remain largely unexplored.

The objectives of this study were to (i) obtain near-chromosome-scale genome assemblies for 18 isolates representative of diverse regions across North America and (ii) to perform a comprehensive comparative genomic analysis to better understand the genomic basis of adaptability of *S. musiva*.

## Methods

### Fungal cultures, nucleic acid extraction and sequencing

*S. musiva* isolates were isolated from infected poplar trees and stored as plugs on K-V8 agar [35] in 50% glycerol at –80 °C [28]. Isolates were regrown in K-V8 liquid broth for 7 days and strained through Miracloth™ (Sigma) to remove the filtrate. The mycelium was frozen in liquid nitrogen. The mycelium was ground by a mortar and pestle to a powder using liquid nitrogen. The Qiagen Genomic-tip 20/G kit with the default protocol was used to extract high-molecular-weight genomic DNA from each sample. DNA was quantified using a Qubit 4 broad range DNA assay kit (ThermoFisher Scientific), and its quality was measured with a NanoDrop One^C^ instrument (ThermoFisher Scientific) based on the 260/280 and 260/230 ratios.

To prepare Oxford Nanopore Technologies (ONT) libraries, DNA was first size selected using a 0.4× ratio of SPRI bead volume to DNA volume. Briefly, ultrapure, molecular-grade water was used to bring DNA volumes up to 200 µl. Then, 80 µl of Ampure XP (Beckman Coulter) beads was added and gently resuspended into the diluted DNA, and this suspension of DNA and beads was incubated for 10 min at room temperature. After incubation, the beads and DNA were pelleted on a magnetic rack and washed three times with 1,000 µl of 80% ethanol. After the pellets were washed and dried, they were resuspended in 30 µl of ultrapure water and incubated at room temperature for an additional 20 min. After the beads were pelleted again, the eluted DNA was retrieved and quantified using the Qubit 4 DNA broad range assay kit (ThermoFisher Scientific). The Native Barcoding Kit 24 V14 was used to prepare long-read ONT libraries following the default protocol. Multiplexed libraries were sequenced on a PromethION 2 Solo device using PromethION flow cells (R10.4.1) and the ONT MinKnow software (v. 23.07.5). Dorado v. 0.7.3 was used to perform basecalling using the dna_r10.4.1_e8.2_400bps_sup@v5.0.0 model (https://github.com/nanoporetech/dorado/).

Preparation and sequencing of Illumina libraries were outsourced to the Oregon State University Center for Quantitative Life Sciences (CQLS). Libraries were prepared using a Wagergen DNA library prep kit and sequenced on an Illumina HiSeq 3000 (Illumina) as 2×150 bp paired-end reads [28].

### Genome assembly

NanoPlot v. 1.42.0 was used to characterize the read quality and length distributions of the sequenced nanopore reads [36]. Chopper v. 0.8.0 was used to filter reads shorter than 1 kb or with an average quality of less than 12 [36]. Given variation in assembly completeness, we used three different assemblers. Specifically, Canu v. 2.2, Flye v. 2.9.4 and nextDenovo v. 2.5.2 were used with their default parameters, except for setting the estimated genome size to 30 Mb, to produce genome assemblies [37,39]. RagTag v. 2.1.0 was used to scaffold contigs into chromosomes based on the MN-14 reference genome [40]. The MUMmer v. 3.1 tool NUCmer was used to align scaffolds with the genome of isolate MN-14 to identify contigs scaffolded in the wrong orientation [41]. RagTag was used again in a gap-filling step for the assembled scaffolds, utilizing alignments of preliminary genome assemblies produced with Flye and nextDenovo that spanned entire gaps as patches. Medaka v. 2.0.0 (https://github.com/nanoporetech/medaka) was used to polish genome assemblies based on the nanopore reads mapped with minimap2 v. 2.28 [42]. In a second round of polishing with Polypolish v. 0.6.0, genome assemblies were polished with Illumina paired-end reads, except for isolates BC-01 and OH-1 for which Illumina reads were not available [43]. In preparation to run Polypolish, the BBmap v. 39.8 tool bbduk.sh was used to trim Illumina reads, which were then mapped to the genome assemblies with BWA mem v. 0.7.18 [44,45].

### Repetitive DNA annotation

RepeatModeler v. 2.0.5 with parameter ‘*-LTRStruct*’ was used to obtain repeat libraries for each assembly [46]. RepeatMasker v. 4.1.5 with parameters ‘*-xsmall -gff -s -a*’ was used to mask repeats in each genome. The R package lme4 was used to fit a linear mixed-effects model on the size and repetitive DNA content of chromosomes. The R package lmerTest was then used to obtain *P*-values for fixed effects using Satterthwaite’s approximation. The script *parseRM.pl* (https://github.com/4ureliek/Parsing-RepeatMasker-Outputs) was used with parameters ‘*--land 50,1*, *--parse* and *--nrem*’ to estimate divergence of repeat families. The RIPper web server was used to identify genomic regions affected by repeat-induced point (RIP) mutations [47]. BEDtools v. 2.31.1 was used to estimate the extent of interspersed repeats overlapping with RIP-affected windows [48].

### Gene prediction

The BRAKER3 pipeline v. 3.0.8 was used with parameter ‘*--fungus*’ and the predictors GeneMark and AUGUSTUS to perform annotation of the soft masked genomes [49,51]. Protein evidence was provided to BRAKER3 as a large database containing 1,778,643 protein sequences from 54 annotated fungal genomes of the class Dothideomycetes, obtained from the NCBI. Liftoff v. 1.6.3 was used with parameters ‘*-polish -exclude_partial -copies -sc 0.90*’ to map gene models from *S. musiva* SO2202 to 18 other genomes to minimize the number of possible genes missing in the annotation [52]. BEDtools v. 2.31.1 was used to identify gene models that did not overlap with current models predicted with BRAKER3 and to identify gene models with more than 50% overlap with interspersed repeats to be removed as predicted transposable elements. AGAT v. 1.4.1 was used to obtain gene annotation statistics, e.g. mean size of genes and introns, and to identify gene models with in-frame stop codons, which were removed [53]. BUSCO v. 5.7.1 and the reference Dothideomycetes 2024-01-08 database were used in protein mode to estimate gene completeness [54].

### Homology-based functional annotation of genes

blastp searches (e-value <1e-10) against the UniProt/Swiss-Prot database downloaded on 03 November 2024 were used to obtain functional descriptions for each gene [55]. InterProScan v. 5.69–101.0 was used to obtain conserved domains for the protein sequences [56]. PANNZER was used to annotate Gene Ontology (GO) terms [57]. GO terms with a positive predictive value smaller than 0.4 were filtered out. DbCAN3 webserver was used to predict carbohydrate-active enzymes (CAZymes) based on HMMdb release 13.0 [58]. blastp searches (e-value <1e-10) were used to predict transporters and proteases based on the databases TCDB version of 10/21/2024 and MEROPS v. 12.5, respectively [59,60]. AntiSMASH v. 7.1.0 was used with relaxed strictness to predict genes involved in secondary metabolite biosynthesis [61]. The clinker web server was used to visualize homology of secondary metabolite gene clusters [62]. SignalP v. 6.0, TargetP v. 2.0 and Phobius v. 1.01 were used to identify predicted secreted proteins [63,65]. Proteins with a positive signal for secretion by at least two of these tools were considered for downstream analyses. TMHMM2.0 and NetGPI v. 1.1 were used to predict genes encoding mature secreted proteins with a transmembrane domain or a GPI anchor [66,67]. These proteins were disregarded for effector prediction. EffectorP3 was used to predict candidate effectors [68]. Mature proteins with less than 200 aa and at least 2% cysteine residues were also classified as candidate effectors.

### Large-scale chromosomal variations

The MCscan pipeline within the JCVI utility libraries (https://github.com/tanghaibao/jcvi) was used to obtain pairwise synteny plots based on pairwise gene homology [69]. The coordinates of the first and last genes of each inversion were used to estimate the size of inversions and to merge them when present in multiple isolates. Minimap2 v. 2.28 and CuteSV v. 2.1.1 were used with parameters *‘--max_cluster_bias_INS 100 --diff_ratio_merging_INS 0.3 --max_cluster_bias_DEL 100 --diff_ratio_merging_DEL 0.3 min_size 50 --max_size 700000 min_support 10 min_read_len 5000*’ to identify the presence of inversions based on ONT reads [70].

### Gene-based pangenome

OrthoFinder v. 2.5.5 was used to organize protein sequences into hierarchical orthologous groups (HOGs) [71]. HOGs containing genes from 95% of the isolates were considered core. Non-core HOGs were considered accessory if containing genes from multiple isolates, or isolate-specific (i.e. singleton) if containing genes from a single isolate. The R package topGO v. 2.56.0 using the ‘*weight01*’ algorithm and Fisher’s exact statistical test was used to perform GO enrichment of accessory HOGs [72]. The R function *p.adjust* was used to adjust the *P*-values based on the false discovery rate (FDR) method used on the total count of GO terms. The nonlinear least squares *nls* function within R was used to obtain the core and pangenome curves by fitting the equation *aN^b^* based on the median values of pan and core genomes, where *N* is the number of genomes analysed, *a* is a scaling constant, and *b* is an exponent determining the rate of gene accumulation [73].

### Phylogenetic tree

MAFFT v. 7.526 was used with parameters ‘*--maxiterate 1000 --localpair*’ to align coding sequences of single-copy BUSCO genes conserved within ascomycetes [74]. TrimAl v. 1.4 as used with parameters ‘*-gt 0.5 -cons 50*’ to trim alignments [75]. IQTREE v. 2.3.4 with parameter ‘*-B 1000*’ was used to infer a maximum likelihood phylogenetic tree [76], which was visualized using ggtree v. 3.12.0 [77].

### Genome compartmentalization analysis

BEDtools v. 2.31.1 was used to obtain upstream and downstream intergenic regions of genes, and their sizes were visualized in hexagonal heatmaps produced with ggplot2 v. 3.5.1 [78]. Genes with either up- or downstream intergenic sizes greater than 10 kb or 5 kb were considered to be in gene-sparse regions. Hypergeometric tests were used to perform enrichment of candidate effectors and accessory genes within gene-sparse regions. Differences in genome compartmentalization were investigated by comparing the mean and median total intergenic sizes of genes. Wilcoxon rank-sum tests were used to test for significant differences in intergenic sizes. The resulting *P*-values were adjusted based on the FDR method in the function *adjust_pvalue* from the R package rstatix v. 0.7.2.

### Gene expression

RNA-seq reads of *S. musiva* isolate MN-14 during interaction with *P. trichocarpa* [32] were obtained from the NCBI SRA database. The BBMap tool bbduk.sh was used with parameters ‘*ktrim=r k=23 mink=11 hdist=1 tpe tbo qtrim=rl trimq=10 minlen=4*’ to trim reads for quality. STAR v. 2.7.11b was used to map trimmed reads to the genome of isolate MN-14 [79]. featureCounts v. 1.6.3 was used to obtain counts of reads mapped to the genes [80]. A custom R script was used to convert raw read counts to transcripts per million (TPM) values (https://github.com/alexzaccaron/2025_smusiva_chr/tree/main/07_gene_expression).

## Results

### Near-chromosome-level assemblies of 18 representative isolates of *S. musiva*

**Fig. 1.**
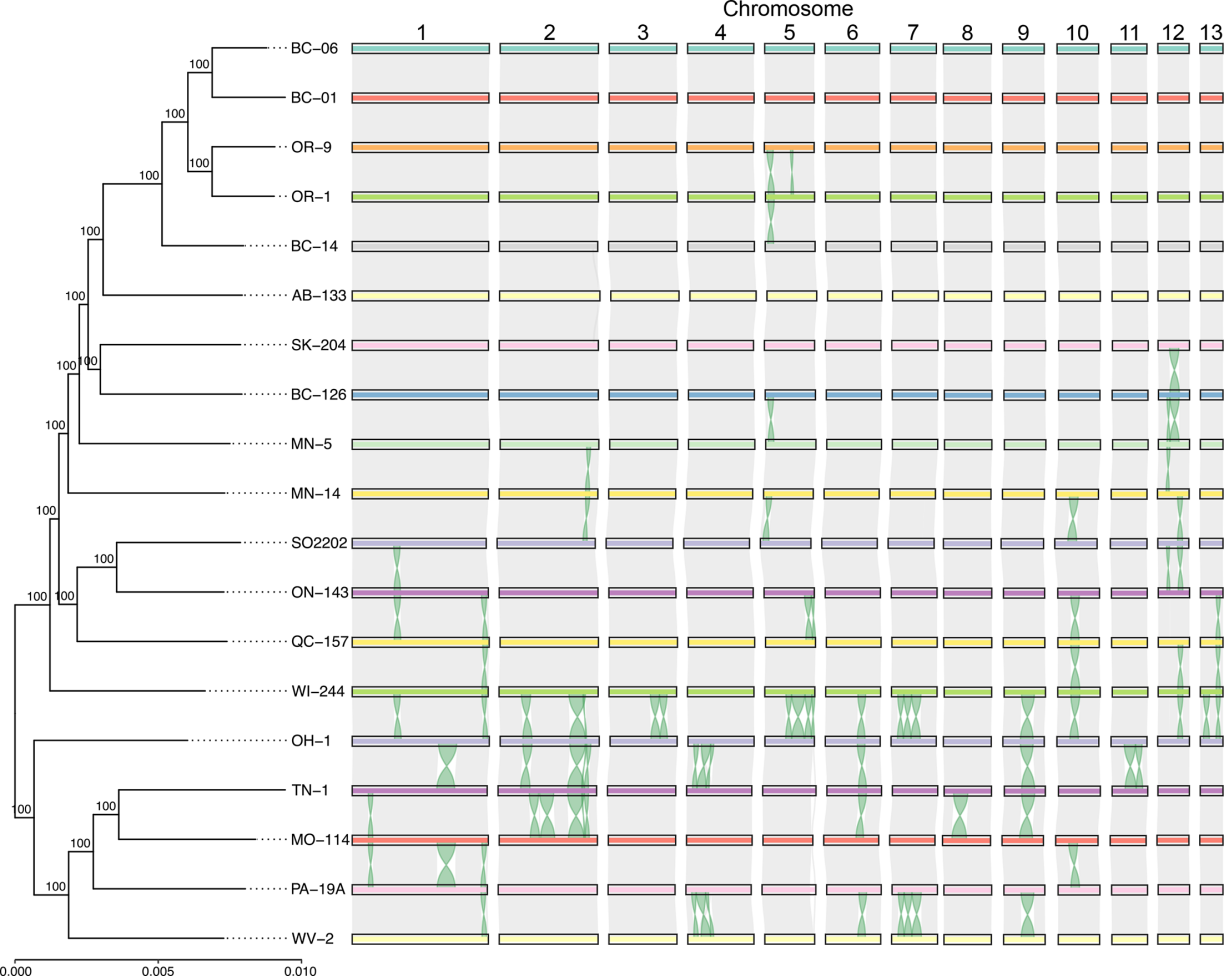
*S. musiva* genomes exhibit numerous, long chromosomal inversions. Phylogeny and synteny of the 13 chromosomes of 19 *S*. *musiva* isolates. The figure shows a maximum likelihood phylogenetic tree constructed based on aligned coding sequences of 1,620 complete single-copy BUSCO genes. Values above branches indicate bootstrap support based on 1,000 ultrafast bootstrap replicates. The tree was rooted at its midpoint. Orthologue-guided chromosome alignments are shown on the right-hand side. Grey bars connecting chromosomes represent regions of synteny. Ribbons representing predicted inversions are highlighted in green.

We selected 18 *S*. *musiva* isolates representative of clades from the USA and Canada for ONT sequencing (Table 1) [28]. We generated between 57× and 264× coverage of long reads per isolate, with read N50 between 3.2 and 19.9 kb (Tables 1 and S1). In addition to ONT reads, we generated between 46× and 69× coverage of Illumina reads per genome for polishing long-read assemblies. Preliminary assemblies were generated for the 18 isolates using the Canu, Flye and nextDenovo assemblers. No single assembler produced the best results across all isolates. However, nextDenovo frequently produced fewer contigs, whereas Flye frequently produced better L90/N90 values (Table S2). Alignments of these assemblies to the genome of isolate SO2202 [30] indicated that Flye and nextDenovo assemblies contained 11 and 30 cases of chimeric contigs formed by sequences from different chromosomes, respectively (Fig. S1, Table S2). In contrast, we detected only one chimeric contig in all Canu assemblies. Thus, we selected the Canu assemblies for downstream analyses. Following a scaffolding step, all 18 sequenced isolates contained 13 chromosomes (chr1-chr13) (Fig. 1). This is consistent with the genome of isolate SO2202, identified by cytological karyotyping 13 chromosomes [30]. Notably, the genome assembly of isolate MN-14 was gapless with no unplaced contigs. The assemblies of the other 17 genomes contained between 3 and 61 small unplaced contigs with an average size of 27.4 kb (Table S3). Between 72% and 100% of the total bases from unplaced contigs matched with at least 95% identity to sequences from the 13 chromosomes of the 19 isolates analysed, suggesting that unplaced contigs represent improperly assembled repetitive sequences from the 13 chromosomes.

**Table 1.** Background data, read coverage and N50 for isolates of *S. musiva* selected for long-read sequencing

Isolate	Origin	Mating type	Genome coverage (X)	Read N50 (bp)
AB-133	Canada, Alberta	MAT1-1	125	6,059
BC-01	Canada, British Columbia	MAT1-2	181	6,516
BC-06	Canada, British Columbia	MAT1-2	91	7,226
BC-126	Canada, British Columbia	MAT1-1	137	6,107
BC-14	Canada, British Columbia	MAT1-2	260	6,032
MN-14	USA, Minnesota	MAT1-2	110	19,911
MN-5	USA, Minnesota	MAT1-1	83	4,174
MO-114	USA, Missouri	MAT1-2	212	5,109
OH-1	USA, Ohio	MAT1-2	110	4,997
ON-143	Canada, Ontario	MAT1-2	57	5,475
OR-1	USA, Oregon	MAT1-1	111	4,471
OR-9	USA, Oregon	MAT1-1	264	6,311
PA-19A	USA, Pennsylvania	MAT1-1	85	6,623
QC-157	Canada, Quebec	MAT1-1	74	6,046
SK-204	Canada, Saskatchewan	MAT1-1	114	9,839
TN-1	USA, Tennessee	MAT1-1	66	5,585
WI-244	USA, Wisconsin	MAT1-1	121	3,254
WV-2	USA, West Virginia	MAT1-2	83	8,042

Among the 18 assemblies plus isolate SO2202, all chromosomes except for chr10 contained predicted telomere sequences at each end in at least one of the assemblies (Fig. S2). The size of the assembled chromosomes varied considerably among the 19 *S*. *musiva* isolates (Fig. S3a, Table S4). The coefficient of variation for the size of the 13 chromosomes ranged between 0.03 and 0.08 (Fig. S3c). However, the variation in size reduced drastically when considering only unmasked bases (i.e. non-repetitive DNA) of the assemblies (Fig. S3b), with coefficients of variation between 0.007 and 0.05 (Fig. S3c). These results indicate that the variation in chromosome size among *S. musiva* isolates is primarily explained by the presence of repetitive DNA.

### Accumulation of repetitive DNA explains the differences in the size of *S. musiva* genomes

**Fig. 2.**
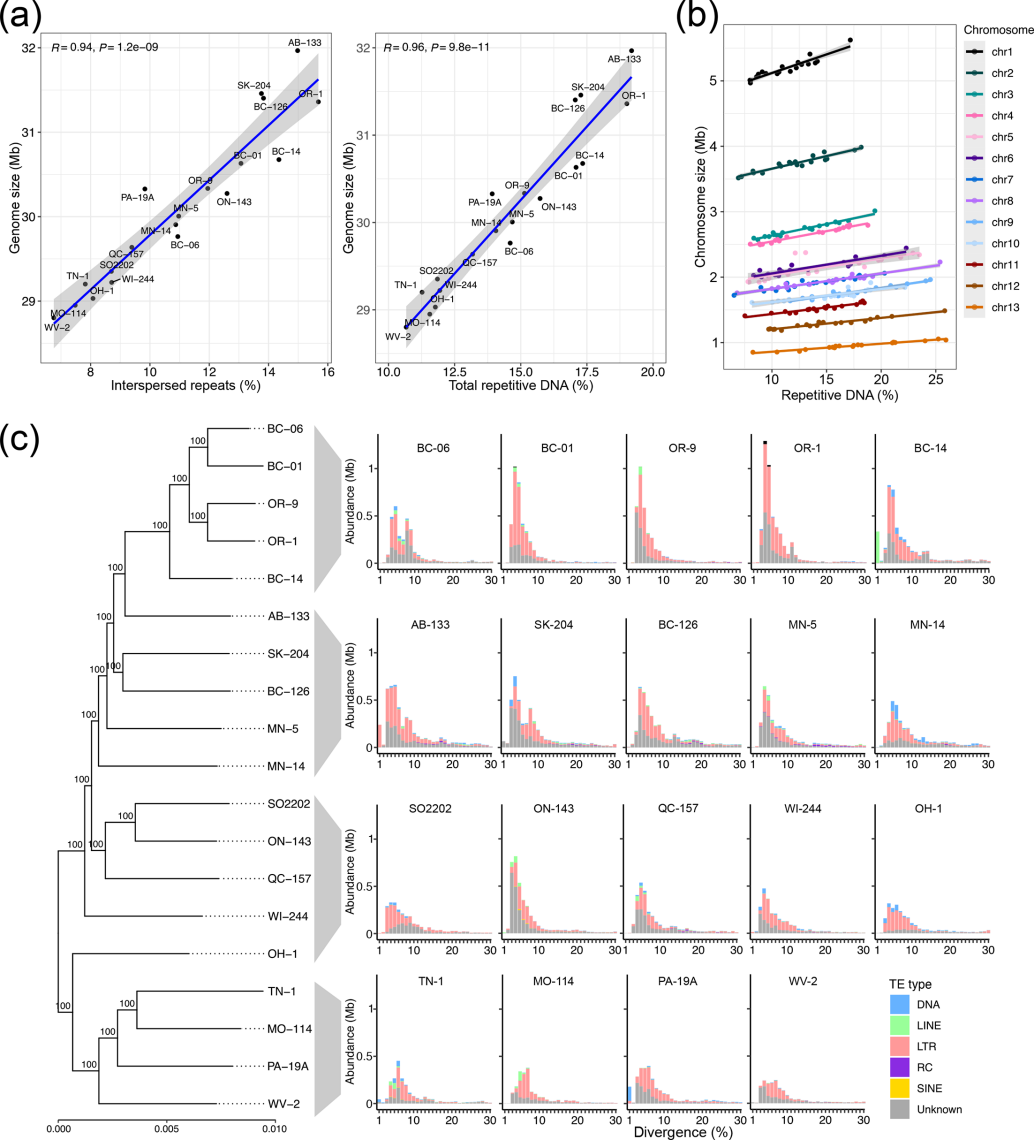
Repetitive DNA content and divergence patterns vary among the genomes of 19 *S. musiva* isolates. (**a**) Scatter plots showing correlation between size of genome assemblies and percentages of total repetitive DNA content and interspersed repetitive DNA content. Shaded area represents 95% confidence intervals. (**b**) Relationship between chromosome size and repetitive DNA content across the 19 isolates. Points represent chromosomes from each isolate, coloured by chromosome identity. Solid lines show chromosome-specific linear regression trends. (**c**) Divergence of repetitive DNA represented as bar plots showing the number of bases covered by predicted TEs from different (sub)classes. Isolates are displayed in the order they appear in the BUSCO gene phylogenetic tree. The tree was rooted at its midpoint.

A *de novo* annotation of repetitive DNA revealed large differences in repeat content among the 19 analysed *S. musiva* genomes. Total bases masked in the genome assemblies ranged from 10.7% to 19.2% (Table 2). Similarly, the total amount of interspersed repeats, i.e. predicted transposable elements (TEs), in the genomes varied from 6.8% to 15.7% (Tables 2 and S5). As expected, there was a positive correlation (*R*=0.94) between the amount of predicted TEs and genome size, and a stronger correlation (*R*=0.96) when considering all repeats in the genomes (Fig. 2a). Moreover, a linear mixed-effects model (Size ~ % Repeats + [1|Chromosome] + [1|Isolate]) revealed a significant positive relationship, with each 1% increase in repetitive DNA corresponding to a 24,960±912.5 bp increase in chromosome size (*t*_88.4_ = 27.35, Satterthwaite-adjusted *P*<2×10^−16^) (Fig. 2b). The G+C content of the sequenced genomes has a bimodal distribution with peaks at 43 mol% and 53 mol% (Fig. S4). The broadly bimodal pattern was more evident in genomes with higher repetitive DNA content. The occurrence of RIP mutations may explain this pattern. RIP mutations induce transition nucleotide substitutions (C-to-T or G-to-A) in repetitive DNA, thus reducing the overall G+C content in repetitive regions of the genome [81]. The percentages of the genomes predicted to be affected by RIP mutations ranged from 10.5% to 18.2% (Table S6). The percentage of RIP mutations was strongly correlated (*R*=0.98) with the abundance of repeats (Fig. S5a). Interestingly, the percentages of repeats affected by RIP also varied considerably, from 81.9% to 98.0%, exhibiting a weak correlation (*R*=0.13) with the abundance of repeats (Fig. S5b). This indicates that some TEs are escaping or have not yet been affected by RIP mutations in some isolates.

**Table 2.** Genome assembly statistics and repetitive DNA content of 18 *S. musiva* isolates compared to the previous reference genome of isolate SO2202

Isolate	Assembly size (bp)	Contig	Scaffold	L50	L90	G+C (mol%)	Interspersed repeat (%)	Simple repeat (%)	Masked base (%)
AB-133	31,966,893	36	35	5	12	50.6	15.0	2.5	19.2
BC-01	30,631,289	28	27	5	11	50.8	13.1	2.6	17.1
BC-06	29,764,371	23	23	5	11	50.9	10.9	2.7	14.6
BC-126	31,403,112	28	28	5	11	50.7	13.8	2.6	17.1
BC-14	30,675,870	25	25	5	11	50.8	14.3	2.6	17.3
MN-14	29,905,407	14	14	5	11	50.9	10.9	2.7	14.1
MN-5	30,006,104	16	16	5	11	50.9	11.0	2.7	14.7
MO-114	28,950,486	21	21	5	11	51.2	7.5	2.8	11.6
OH-1	29,031,431	19	18	5	11	51.2	8.1	2.7	11.8
ON-143	30,274,891	49	45	5	11	50.8	12.6	2.6	15.7
OR-1	31,360,006	85	74	5	12	50.5	15.7	2.6	19.0
OR-9	30,334,013	25	25	5	11	50.9	11.9	2.7	15.1
PA-19A	30,327,956	51	48	5	12	51.1	9.8	2.6	13.9
QC-157	29,637,038	18	18	5	11	50.9	9.4	2.7	13.2
SK-204	31,458,548	17	17	5	11	50.7	13.8	2.7	17.3
TN-1	29,201,187	27	25	5	11	51.1	7.8	2.8	11.3
WI-244	29,221,326	35	32	5	11	51.1	8.7	2.8	11.9
WV-2	28,801,464	17	16	5	11	51.2	6.8	2.7	10.7
SO2202	29,352,103	458	72	5	11	51.1	8.7	2.8	11.8

We characterized the abundance of different TE categories in the sequenced genomes (Fig. 2c, Table S5). Of the different classes of repeat, the abundance of long terminal repeat (LTR) elements varied the most across isolates. The LTR content of each genome ranged from 29.6% to 51.1% of the masked bases (Table S5). The abundance of DNA transposon elements also varied considerably, ranging from 0.8% to 12.8% of the masked bases. The abundance of unclassified TEs ranged from 14.9% to 43.7% of the masked bases. We characterized the overall divergence of predicted TEs in the *S. musiva* genomes and identified an abundance of TEs with 3%–6% divergence, similar to previous findings [31]. Interestingly, TEs with a predicted 1% or less divergence were abundant in only a few isolates: BC-14, AB-133 and PA-19A. In the genome of isolate AB-133, nearly all bases covered by these predicted low-divergence TEs were classified as LTR elements, consistent with previous reports for a lineage of *S. musiva* from Alberta, Canada [31]. In contrast, nearly all bases covered by predicted low-divergence TEs in isolate BC-14 were classified as long interspersed nuclear elements, and in PA-19A, most low-divergence TEs were predicted to be DNA transposons. These results indicate that *S. musiva* underwent lineage-specific expansion of TEs.

### *S. musiva* has a stable gene content

*De novo* gene prediction identified between 9,764 and 9,933 protein-encoding genes among the 18 *S*. *musiva* genomes, with BUSCO completeness of at least 98.9% and less than 1% missing genes (Table 3, Fig. S6). In comparison, 10,144 genes were predicted in the previous reference genome of isolate SO2202, which had a lower BUSCO completeness of 97.7%. Homology-based functional annotation assigned GO terms to 7,045 to 7,177 genes per isolate and between 6,922 and 7,034 genes contain conserved PFAM domains (Table S7). Moreover, similar numbers of genes from different functional categories were observed across the genomes. Among the 18 annotated genomes, there are 361–369 genes encoding CAZymes, 285–292 proteases, 220–226 major facilitator superfamily (MFS) transporters, 39–41 ATP-binding cassette (ABC) transporters, 30–34 key genes for secondary metabolism biosynthesis, 970–997 secreted proteins and 319–341 candidate effectors (Table S7).

**Table 3.** Gene prediction statistics of the 18 *S. musiva* isolates sequenced compared to the previous reference genome of isolate SO2202

Isolate	Gene count	Average size			BUSCO completeness (%)			
		Gene (bp)	Exon (bp)	Intron (bp)	Protein (aa)	Complete	Single copy	Missing
AB-133	9,840	1,594.4	656.5	90.8	489.8	99.0	98.4	0.7
BC-01	9,795	1,589.7	656.2	90.4	488.7	99.0	99.0	0.8
BC-06	9,817	1,589.8	655.6	90.8	488.5	99.0	98.9	0.8
BC-126	9,813	1,593.0	656.2	90.3	489.7	99.0	99.0	0.7
BC-14	9,787	1,591.2	655.4	90.6	489.0	99.0	99.0	0.8
MN-14	9,807	1,592.7	657.3	89.7	489.8	99.0	99.0	0.8
MN-5	9,789	1,593.5	657.6	90.4	490.0	99.2	99.2	0.6
MO-114	9,767	1,595.5	657.2	89.8	491.2	99.1	99.1	0.6
OH-1	9,785	1,592.5	656.8	89.8	489.9	99.0	98.9	0.7
ON-143	9,844	1,592.3	654.0	91.4	488.6	99.1	99.1	0.7
OR-1	9,839	1,590.0	655.8	91.0	488.9	98.9	98.7	0.9
OR-9	9,776	1,591.5	655.8	90.1	489.5	99.0	99.0	0.8
PA-19A	9,933	1,595.1	656.2	90.9	490.5	99.0	97.7	0.8
QC-157	9,801	1,593.1	656.7	90.3	489.7	98.9	98.9	0.8
SK-204	9,837	1,589.5	655.2	90.3	488.7	99.1	99.0	0.6
TN-1	9,836	1,595.5	656.0	90.4	490.8	99.1	99.0	0.8
WI-244	9,799	1,596.8	656.8	90.7	490.5	99.0	99.0	0.7
WV-2	9,764	1,597.8	657.2	90.2	491.6	99.1	99.1	0.7
SO2202	10,144	1,997.6	753.4	116.7	451.5	97.7	97.7	1.2

**Fig. 3.**
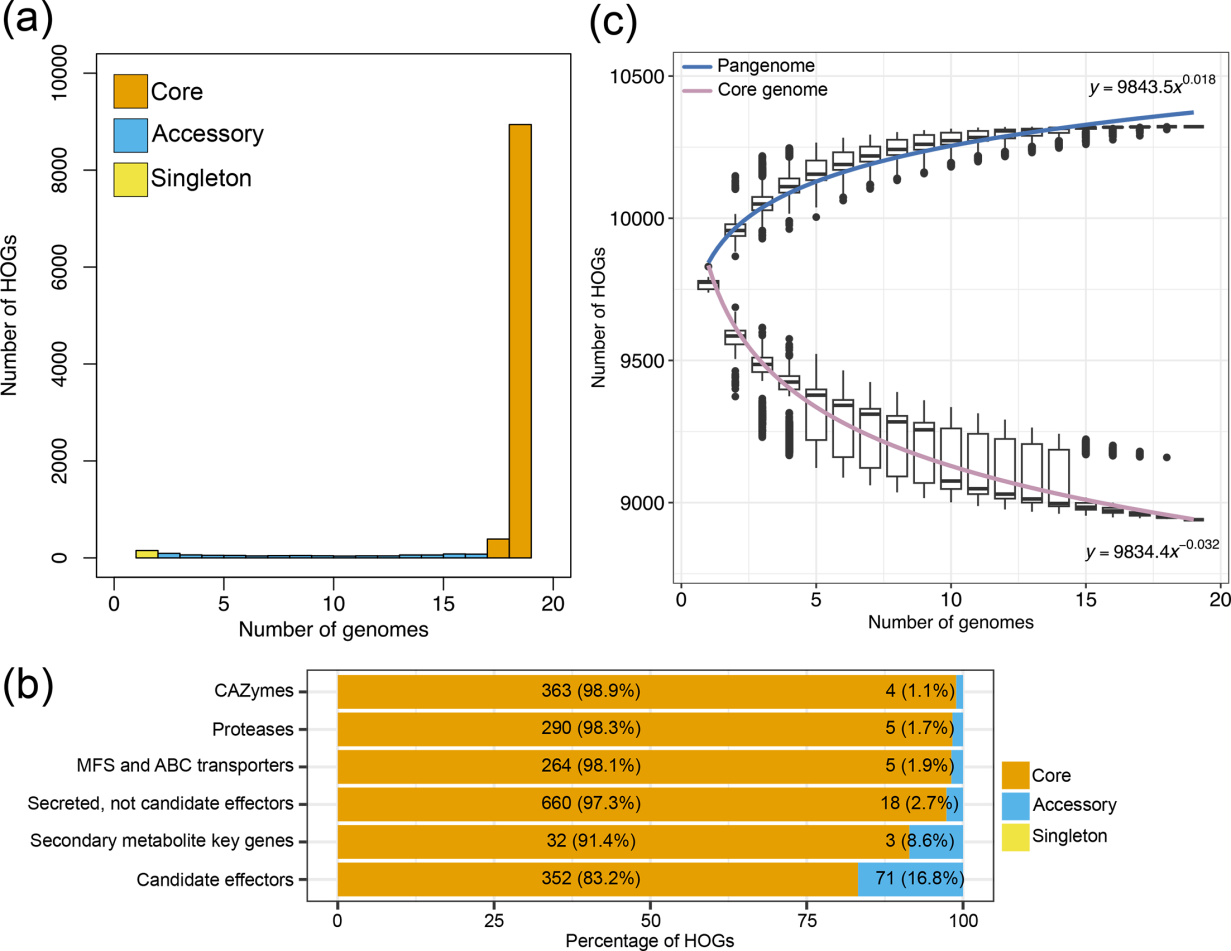
*S. musiva* has a closed pangenome, but variation in effector gene content. (**a**) Histogram showing the distribution of the number of core (present in 95% of the genomes), accessory and singleton (i.e. represented by only one genome) hierarchical orthogroups (HOGs). (**b**) Bar plot showing counts and percentages of core and accessory HOGs containing genes encoding CAZymes, proteases, transporters of the major facilitator and ABC families, secreted proteins not classified as candidate effectors, key genes for secondary metabolite biosynthesis and candidate effectors. (**c**) Scatterplot showing the estimated sizes of pan- and core genome of *S. musiva*. The 19 genomes were sampled in all possible combinations of size *N*, with 1 ≤ *N* ≤ 19. Points represent the number of all HOGs (pangenome) and HOGs containing genes from all sampled genomes (core genome). The pan- and core-genome accumulation curves are represented by a least squares fit of the power law *n*=*aN^b^*, where *n* is the predicted pangenome size, *N* is the number of genomes sampled (1 ≤ *N* ≤ 19) and *a* and *b* are constants.

A gene-based pangenome was constructed for the 18 sequenced isolates of *S. musiva* plus the previous reference genome of isolate SO2202. The protein sequences from all predicted genes were clustered into a total of 10,322 HOGs (Table S8). Of these, 8,940 (86.6%) contained genes present in all 19 isolates analysed (Fig. 3a). When considering HOGs absent in at most one isolate (i.e. present in 95% of the isolates), the number of HOGs increased to 9,329 (90.3%). We considered these as core orthologous groups in *S. musiva*. The remaining 993 HOGs were classified as either accessory (*n*=980; 9.5%) or isolate-specific (*n*=13; 0.1%). A GO enrichment analysis of accessory HOGs indicated significant enrichment of only two terms: mycotoxin biosynthetic process (GO:0043386; FDR-adjusted *P*-value=0.003) and membrane (GO:0016020; FDR-adjusted *P*-value=1.1e-6). This indicates a high abundance of accessory genes involved in secondary metabolism biosynthesis and proteins acting on cell membranes.

A pangenome accumulation curve supported a stable gene complement among *S. musiva* isolates (Fig. 3c). By obtaining all possible subsets of one to 19 genomes, the median size of the pangenome ranged from 9,775 to 10,273. However, it rapidly stabilized when subsets of 15 or more genomes were included, for which the median size of the pangenome varied slightly from 10,319 to 10,322. The pangenome accumulation curve fitted the power law regression formula *y=axᵇ* with the exponent *b*=0.018, which is indicative of a closed pangenome [73].

Further analysis of functional gene categories revealed low percentages (1%–3%) of accessory HOGs containing genes encoding CAZymes, proteases, MFS and ABC transporters and secreted proteins not classified as effectors (Fig. 3b). These gene categories were significantly depleted among accessory HOGs (hypergeometric test, *P*-value<3e-7). In contrast, candidate effectors were significantly enriched (hypergeometric test, *P*-value=1.1e-6) among accessory HOGs. Specifically, 16.8% of HOGs containing candidate effectors were classified as accessory. Analysis of the presence/absence of candidate effectors in accessory HOGs revealed no obvious association pattern between the phylogeny of isolates and the presence of accessory candidate effectors (Fig. S7). Using RNA-seq data from the interaction of *S. musiva* isolate MN-14 with *P. trichocarpa*, we detected considerable levels of expression (transcripts per million TPM >100) for five accessory candidate effector genes (SMMN14_00967, SMMN14_01600, SMMN14_01567, SMMN14_04983 and SMMN14_06052) in all three replicates of at least one time point during infection (Fig. S8, Table S9). These results suggest that the repertoire of effector genes in *S. musiva* is more dynamic in terms of gene presence/absence compared to other functional gene categories and that some accessory candidate effectors are actively expressed during host colonization.

### Long chromosomal inversions affect over a third of the *S. musiva* genome

**Fig. 4.**
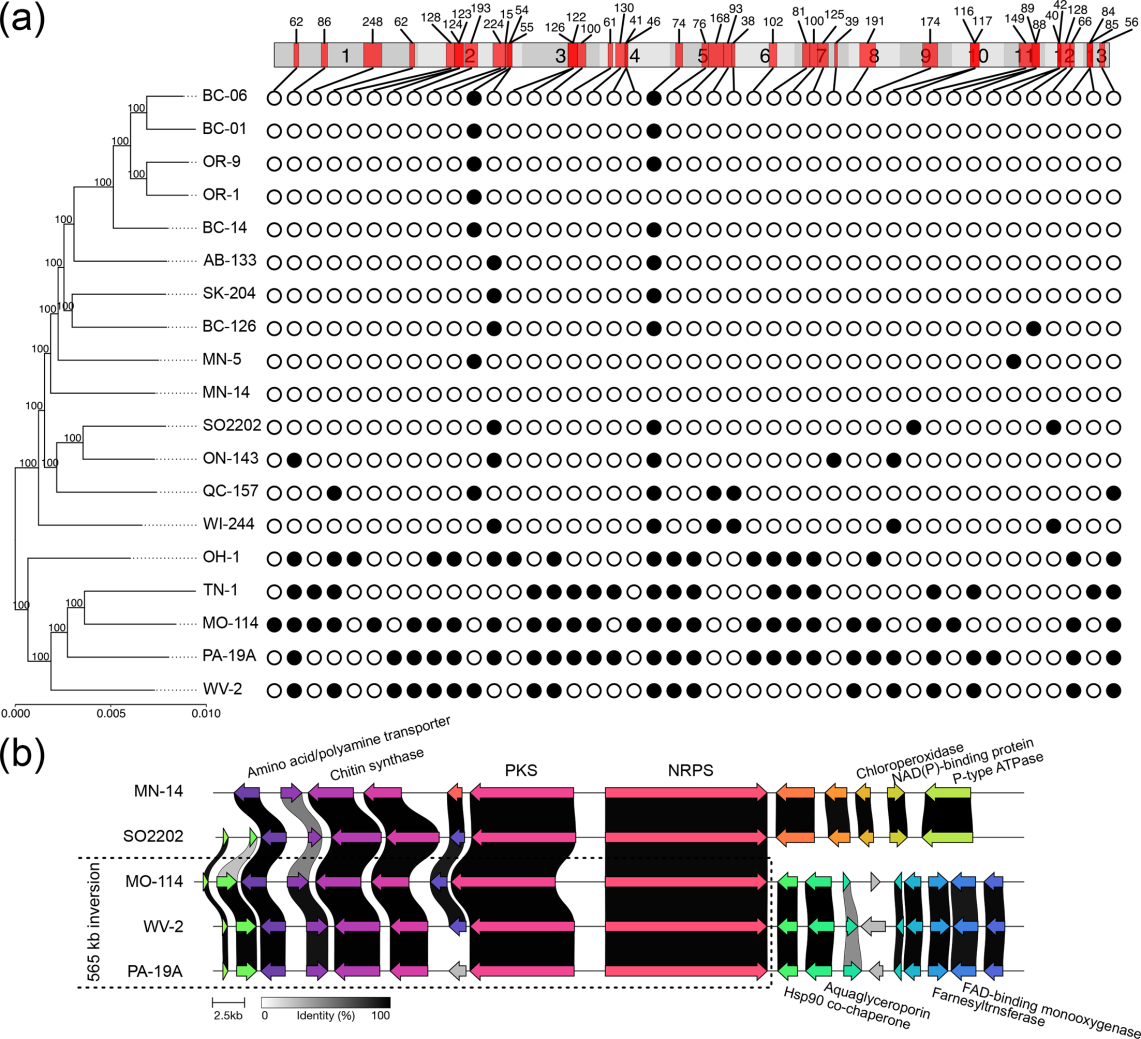
Chromosomal inversions are common in the genome of *S. musiva*. (**a**) Location and size of inversions represented as rectangles on the 13 pseudochromosomes of the reference genome of isolate MN-14. Numbers above the inversions indicate the number of genes within the inversion. Filled and empty circles represent the presence and absence of inversions in the respective isolates. On the left-hand side, a maximum likelihood phylogenetic tree was inferred based on aligned coding sequences of 1,620 complete single-copy BUSCO genes. The tree was rooted at its midpoint. (**b**) Predicted biosynthetic secondary metabolite gene cluster affected by a 565 kb inversion present on the chromosome chr8 of isolates MO-114, WV-2 and PA-19A. The cluster contains two backbone genes encoding a polyketide synthase (PKS) and a nonribosomal peptide synthetase (NRPS). The predicted function or functional description of other genes in the cluster is shown. The location of the inversion breakpoint is downstream of the NRPS gene, as indicated with a dashed line.

A phylogeny-based synteny analysis based on pairs of orthologous genes from the 13 chromosomes revealed one-to-one chromosome homology with no interchromosomal translocations among 19 *S*. *musiva* genomes (Fig. 1). In contrast, the order of genes within the chromosomes varied considerably due to multiple, long chromosomal inversions (Fig. 1). Using the genome of isolate MN-14 as a reference, a synteny analysis revealed a total of 146 inversions, each affecting between 15 and 248 genes. By categorizing inversions affecting the same genes into groups, we identified 43 distinct inversions that varied in presence/absence among the 18 isolates (Fig. 4a, Table S10). Collectively, 10.2 Mb (34.1%) and 3,605 genes (36.8%) of the reference genome of isolate MN-14 were affected by inversions in at least one isolate. Across the 19 isolates, long inversions were detected in all chromosomes, but the extent to which they were affected varied considerably. Chromosome chr5 was the most dynamic, harbouring five long inversions that changed the orientation of 449 (68.2%) of the 658 genes in this chromosome. In contrast, a single inversion detected in chromosome chr6 affected 102 (14%) of the 729 genes.

Interestingly, some inversions were physically close to each other and could be further classified as tandem (*n*=15) if there were no genes in between, proximal (*n*=2) if there were at most ten genes in between or distal (*n*=25) (Table S10). The second half of chr5, for example, was strongly impacted by four tandem inversions with an average size of 293 kb. Further pairwise nucleotide alignments supported the presence of tandem and proximal inversions (Fig. S9), indicating that these are not artefacts produced by the gene-anchored synteny analysis. Moreover, the ONT reads from 17 isolates mapped to the genome of MN-14 supported the presence of 64 out of 142 total inversions among these isolates (Table S11).

The long chromosomal inversions were more frequently observed in isolates from across the eastern-central part of the USA (Fig. 4a). For example, a total of 30 inversions exhibited presence/absence variation among the 5 isolates from WV, PA, MO, TN and OH. In contrast, only 14 inversions exhibited presence/absence variation among the remaining 13 isolates from MN, WI, OR and Canada. This indicates that instead of being widespread among different lineages of *S. musiva*, long chromosomal inversions are more frequently present in isolates from the eastern-central USA, which spans the native area of *S. musiva* [23], and occur more rarely in isolates from the US Northwest and Canada.

### Impact of chromosomal inversions in the *S. musiva* genome

To investigate the potential functional impact of the identified long chromosomal inversions in the *S. musiva* genome, 226 genes flanking the predicted inversion breakpoints (±3 kb) were analysed. These genes were members of 225 HOGs, of which 204 (90.7 %) were classified as core HOGs, while only 21 (9.3%) were accessory. Moreover, only 10 HOGs (4.4%) contained more than one gene per isolate, i.e. including putative duplicated genes. These numbers are similar to those observed for the entire genome, which contained 9.5% accessory HOGs and 3.8% containing putative duplicated genes. These observations indicate that inversion breakpoints are not major contributors to the gain or loss of genes through deletion, pseudogenization, or duplication. A total of 36 breakpoints from 29 inversions were located to promoter-proximal intergenic regions (<1 kb upstream) of one or both genes flanking the breakpoints. This positioning suggests potential regulatory impacts on the expression of adjacent genes. The genes flanking inversion breakpoints are predicted to be involved in a wide range of functions and include 6 CAZymes, 9 proteases, 1 nonribosomal peptide synthetase (NRPS) gene and 12 candidate effectors. Among 21 gene functional categories investigated, only metalloproteases (hypergeometric test *P*-value=0.036) were significantly enriched among genes flanking inversion breakpoints (Table S12).

Interestingly, a 565 kb inversion in chr8 of isolates MO-114, WV-2 and PA-19A affected a predicted secondary metabolite gene cluster. The left-hand side breakpoint of this inversion is in the downstream intergenic region of an NRPS gene that, together with a PKS, forms the backbone of the cluster (Fig. 4b). In the three isolates with this inversion, the five genes downstream of the NRPS, including a predicted chloroperoxidase, a NAD(P)-binding protein and a P-type ATPase, are replaced by nine new genes located on the other side of the inversion breakpoint. Among these nine new genes are four encoding hypothetical proteins, a FAD-binding monooxygenase, a farnesyltransferase, an aquaglyceroporin, a Hsp90 co-chaperone and a protein with a conserved domain similar to haem-dependent oxidative N-demethylase (Table S13). These results reveal that inversions in the *S. musiva* genome not only altered gene order but also promoted the origin of a putative new secondary metabolite gene cluster by replacing genes encoding putative tailoring enzymes. Interestingly, while almost all genes from this cluster had homologs in closely related species of the family Mycosphaerellaceae, the most similar proteins to those encoded by the PKS and NRPS backbone genes were from species outside the class Dothideomycetes, suggesting that these two genes may have been acquired horizontally or lost in closely related species (Table S13).

To further investigate the impact of inversions in the *S. musiva* genome, all 3,605 genes predicted to be within inversion regions were analysed. Among them are 139 CAZymes, 117 proteases, 737 transporters, 121 candidate effectors and 14 key genes for secondary metabolite biosynthesis (Table S14). However, no GO term was found to be enriched (FDR-adjusted *P*-value<0.05) among the 3,605 genes, indicating that inversions can alter the orientation of a large number of genes involved in a wide array of functions. On the other hand, an analysis of 21 distinct functional gene categories indicated that, among the 3,605 genes affected by inversions, there was significant enrichment of polyketide synthase (PKS) and polysaccharide lyase (PL) genes (hypergeometric test *P*-value<0.05) (Table S15). Specifically, six out of the eight predicted PKS genes and five out of the six predicted PL genes in the reference genome of isolate MN-14 were within inverted regions in at least one of the other analysed genomes.

### The genome of *S. musiva* exhibits small-scale compartmentalization

While chromosomal inversions can significantly impact genome organization, the uneven distribution of repetitive DNA also plays a crucial role in shaping the genomic structure of some fungal pathogens. This uneven distribution can produce a compartmentalized architecture composed of gene-poor regions interspersed with gene-rich regions [6,8]. Typically, gene-rich regions are poor in repetitive DNA, while gene-poor regions are rich in repeats and candidate effector genes [6,7]. The considerable differences in TE content among the *S. musiva* isolates reported herein provide a valuable opportunity to investigate variation in genome compartmentalization within a species.

**Fig. 5.**
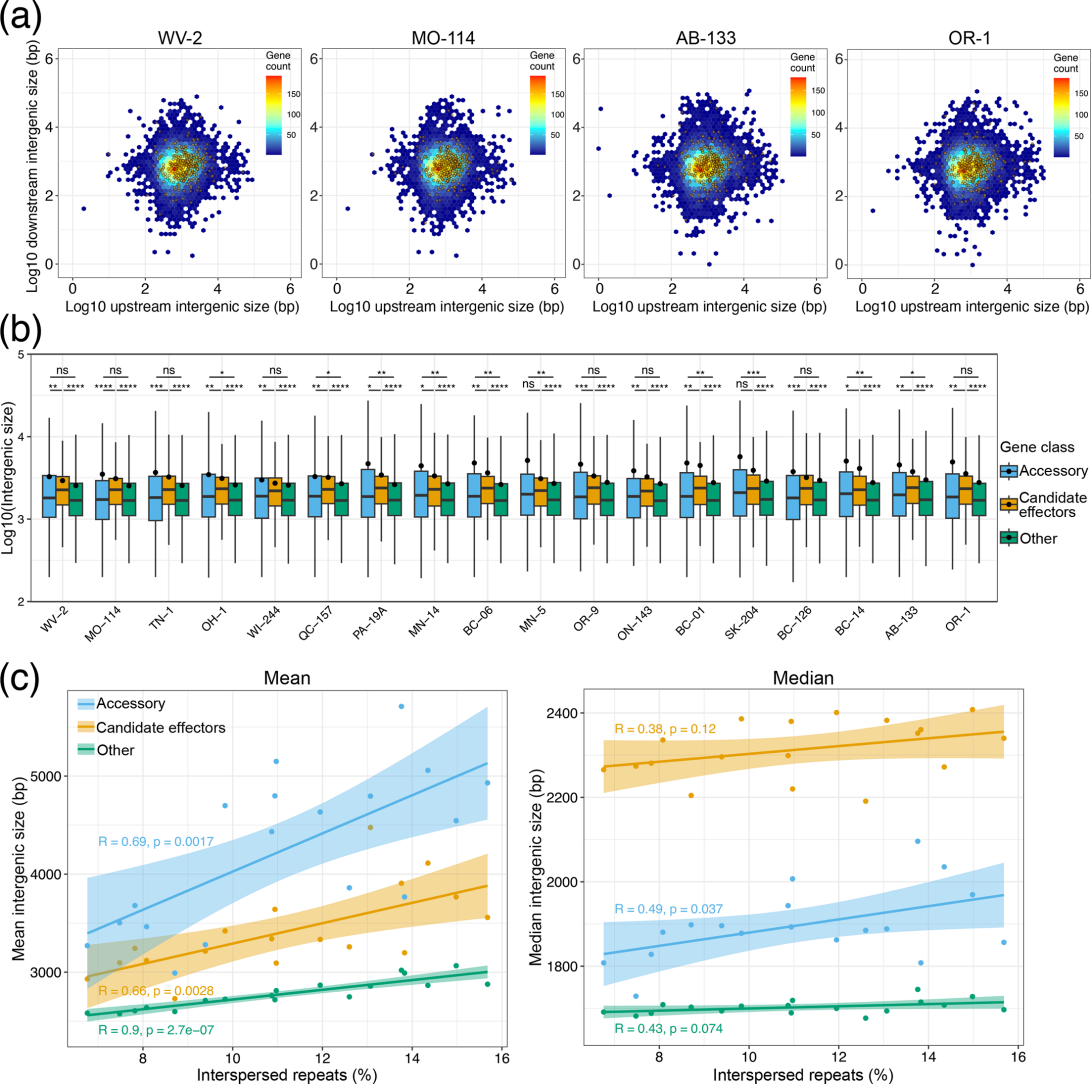
The genome of *S. musiva* shows evidence of small-scale compartmentalization. (**a**) Density plots of intergenic regions of all predicted genes for isolates WV-2 and MO-114 with overall low interspersed repeat content (6.8% and 7.5%, respectively) and isolates AB-133 and OR-1 with overall high interspersed repeat content (15.0% and 15.7%, respectively). Candidate effector genes are shown as transparent points. (**b**) Comparison of the distribution of total intergenic size, i.e. sum of up- and downstream, in log10 scale of accessory genes, candidate effectors and core non-candidate effector genes in 18 isolates of *S. musiva*. Isolates are organized by overall interspersed repeat content, from the lowest on the left-hand side to the highest on the right-hand side. For better visualization, outliers were omitted. Points represent mean values. Statistical significance was inferred by pairwise Wilcoxon rank-sum tests followed by FDR *P*-value adjustment, *P<0.05, **P<0.01, ***P<0.001 and ****P<0.0001. (**c**) Scatter plots showing correlation between percentage of interspersed repeats in the genomes and the mean and median total intergenic sizes of accessory genes, candidate effector genes and core non-candidate effector genes in 18 *S*. *musiva* genomes. Regression lines with 95% confidence interval are shown.

Considering the 18 genomes sequenced in this study, we observed small intergenic sizes with median values ranging from 788 to 815 bp for upstream and 692 to 708 bp for downstream regions (Table S16). In comparison, the average values were higher, ranging from 1,374 bp to 1,672 bp for upstream and 1,208 to 1,424 bp for downstream regions, indicating the presence of a relatively small number of long intergenic regions that skew the average from the median. Indeed, all isolates had between 176 and 356 genes with long intergenic sizes of at least 10 kb either up- or downstream. These were considered genes in gene-sparse regions. Density plots of intergenic region sizes showed no obvious clustering of candidate effector genes in gene-sparse regions (Fig. 5a, Fig. S10). Further analyses indicated that only three isolates (BC-01, BC-06 and BC-14) were enriched for candidate effectors in gene-sparse regions (hypergeometric test *P*-value<0.05). In comparison, 16 out of the 18 analysed genomes had enrichment of accessory genes in gene-sparse regions (hypergeometric test *P*-value<0.05). Similar results were observed when considering 5 kb instead of 10 kb as the threshold to define gene-sparse regions (Table S17). However, in all analysed isolates, candidate effectors had significantly longer intergenic sizes compared to core, non-candidate effector genes (Fig. 5b). The intergenic regions of accessory genes, however, were frequently not significantly longer compared to core, non-candidate effector genes (Fig. 5b). While there is no obvious enrichment of candidate effectors in gene-sparse regions, the existence of longer intergenic regions suggests that the *S. musiva* genome exhibits small-scale compartmentalization.

To investigate changes in genome compartmentalization among genomes with varying TE content, the mean and median sizes of intergenic regions were analysed in relation to the percentage of interspersed repeats. As expected, a positive correlation (*R*=0.9) was observed between mean intergenic size and TE content (Fig. 5c). However, the correlation was weaker (*R*=0.43) and non-significant between the median intergenic size and TE content. The increase in intergenic size of candidate effectors was steeper than that of core non-candidate effector genes (Fig. 5c). For example, when comparing isolates OR-1 and WV-2, which have the highest and lowest interspersed repeat content, respectively, the mean intergenic region of candidate effector genes in OR-1 was 21.4 % longer than in WV-2. In contrast, the mean intergenic region for core non-candidate effector genes was only 9.1 % longer in OR-1 compared to WV-2.

## Discussion

One of the most prominent features reported herein of the *S. musiva* genome is the occurrence of long inversions affecting genes in all 13 chromosomes. Inversions are a major source of genetic diversity and have been associated with phenotypic changes and adaptation in many species. Examples include increased fertility and brain morphology in humans [82,83], speciation of animals [84] and flowering time and seed germination in plants [85]. Notably, the embryo spot trait of potato was recently associated with a 6.49 Mb inversion with a breakpoint occurring in the promoter region of the transcription factor R2R3, affecting its expression [86]. However, the functional impact of inversions in fungi has been less explored. In *Saccharomyces cerevisiae*, inversion of the *DAL2* gene from the DAL gene cluster involved in allantoin metabolism resulted in altering its expression and negatively affecting yeast fitness during nitrogen starvation [87]. In the basidiomycete *Kwoniella*, pericentromeric inversions have been hypothesized to play important roles leading to chromosome fusion, resulting in variable karyotypes with a variable number of chromosomes [88]. By analysing the genomes of 19 *S*. *musiva* isolates, we detected 43 long inversions that covered 34% of the genome. The overall high abundance of inversions observed is in accordance with the hypothesis that fungi from the class Dothideomycetes rapidly accumulate inversions, resulting in the phenomenon known as mesosynteny. This refers to extensive gene shuffling but conserved gene content between homologous chromosomes [89,90]. Our results also indicated that inversions are less frequently observed in *S. musiva* isolates from the Northwestern USA and Canada, whereas they occur more frequently in isolates from the Mideastern USA, which covers the native range of *S. musiva* [23]. It is currently unknown whether there is selection for inversions in *S. musiva*. However, one explanation is that older populations, which are expected to exhibit high diversity, are more likely to accumulate and retain non-deleterious inversions that naturally occur over time.

The overall lack of genes with presence/absence variation adjacent to inversion breakpoints indicates that most of the inversions occurring in *S. musiva* are stable, i.e. they affect the order but not the content of genes, but they might affect regulatory regions of genes, thus affecting their expression. One notable inversion of 565 kb affected the organization of a predicted secondary metabolite gene cluster containing two backbone genes, a PKS and an NRPS, that appeared to have been acquired horizontally. The product of this gene cluster is currently unknown. However, we suggest that this gene cluster in isolates carrying the 565 kb inversion is differentially regulated or produces a chemically different final product due to the putative new genes encoding tailoring enzymes that can introduce new chemical modifications to the core structure of the metabolite. For example, the farnesyltransferase could add a farnesyl group to the metabolite (i.e. farnesylation), altering its lipophilicity and cellular localization [91,92]. Similarly, the FAD-binding monooxygenase could oxidize the metabolite by introducing oxygen atoms at specific sites, creating new functional groups [93].

Our results indicated that *S. musiva* has a closed pangenome, a feature relatively rare among fungal pathogens [94,96]. This genomic characteristic likely reflects *S. musiva*’s specialization to *Populus* species. *S. musiva* also has an overall low percentage (9.5%) of accessory genes. This number contrasts with the estimated 45% and 57% accessory genes in the closely related wheat pathogens *Zymoseptoria tritici* and *Pyrenophora tritici-repentis*, respectively [16,95]. Notably, accessory genes in *S. musiva* were depleted in CAZymes and proteases. This likely reflects a highly conserved repertoire of genes among populations to degrade, modify or create glycosidic bonds, with functions extending from degrading plant cell wall components to fungal cell wall remodelling and nutrient acquisition [97,98]. In contrast, accessory genes were enriched for candidate effectors. This is not surprising, as rapid gain or loss of effectors is frequently observed in fungal pathogens in response to selection pressure exerted by the host [99]. For example, loss of effector genes in *Cladosporium fulvum* isolates is commonly associated with overcoming resistance provided by cognate matching resistance genes in tomato [13]. Conversely, horizontally acquired effector genes provide pathogenicity or increased virulence in *Fusarium oxysporum* [100] and *Bipolaris sorokiniana* [101]. Although our results do not support lineage-specific expansion or contraction of effector genes, one possibility is that *S. musiva* underwent frequent gain or loss of effectors during co-evolution with *Populus* spp.

TEs have profound impacts on fungal genomes, as they can comprise nearly 90% of genomes in some fungal species [102,103]. Their proliferation inflates genome size and can largely explain variations in genome size within and between species [104]. The presence and proliferation of TEs have also been regarded as major evolutionary drivers, leading to genetic diversity associated with adaptation in fungi, such as gain of resistance to fungicides [10,105] and gain of pathogenicity by inducing loss of avirulence genes [11,106, 107]. In our study, we showed that differences in *S. musiva* genome sizes are largely explained by varying amounts of predicted TEs, ranging from 6.8% to 15.7%. Similar variation in TE content has been reported for other fungal plant pathogens, e.g. 4.6%–12.1% in *Magnaporthe oryzae* [108] and 14% and 21.5% in *Z. tritici* [5]. Both species experienced lineage-specific expansion of TEs. In *S. musiva*, higher TE content was observed in isolates from Oregon, British Columbia and Alberta compared to isolates from the southeastern USA. This is in accordance with the previously reported AB lineage from Alberta that had higher TE content [31]. A previous population study predicted three evolutionary lineages of *S. musiva*, corresponding to isolates from British Columbia, the Midwestern USA and the Southeastern USA [28]. Our results presented herein corroborate these findings and further support the hypothesis of lineage-specific expansion of TEs in *S. musiva*. Because the genomes of isolates from the southeastern US were generally less abundant in TEs, we hypothesize that low TE content was the ancestral state of *S. musiva*, although we acknowledge the possibility of TE reduction over time in the southeastern US lineage.

As TEs proliferate in the genomes of fungi and oomycetes, they tend to accumulate in regions that are already rich in TEs, possibly due to selection against insertion in regions more likely to disrupt essential genes [8]. This uneven distribution of TEs throughout the genome can result in a distinctive compartmentalization into gene-rich, repeat-poor regions interspersed with gene-poor, repeat-rich regions. This compartmentalization is the basis for the ‘two-speed genome’ model of evolution reported in many fungal and oomycete pathogens [6,7, 12]. In our analyses, no obvious enrichment of candidate effectors was observed in gene-sparse regions of the *S. musiva* genome, which appears to be in better accordance with the ‘one-speed genome’ model of evolution [7]. However, in all analysed isolates, the intergenic regions of candidate effectors were significantly longer compared to those of core non-candidate effector genes. Although the *S. musiva* genome does not have the traditional architecture of a ‘two-speed genome’, we cannot disregard the possibility that it evolves in a similar manner. For example, the enrichment of candidate effectors among accessory genes could be the result of gene deletion or pseudogenization promoted by the presence of neighbouring TEs.

Among species from the class Dothideomycetes, both ‘one-speed’ and ‘two-speed’ genomes have been reported. For example, the genomes of the grass pathogens *Pyrenophora tritici-repentis* and *Ramularia collo-cygni* exhibit features of ‘one-speed genomes’ [95,109], while the genome of the tomato pathogen *C. fulvum* displays patterns of a ‘two-speed genome’ [110]. The mechanisms mediating contrasting patterns of fungal genome compartmentalization remain unknown. However, a recently proposed hypothesis suggests that the degree of genome compartmentalization is associated with recent bursts of TE proliferation in the evolutionary history of the species [111]. We explored this hypothesis by investigating changes in intergenic size across *S. musiva* isolates with different percentages of TEs in their genomes. Our comparative analysis revealed that the average intergenic size of candidate effector genes increases faster compared to that of core non-candidate effector genes in genomes with higher amounts of predicted TEs. Thus, our results support the hypothesis that fungal genomes become more compartmentalized due to the recent proliferation of TEs.

In summary, by obtaining near-chromosome-scale genome assemblies, we have provided new insights into the organization and structural variations in the genome of the poplar pathogen *S. musiva*. We have shown that the *S. musiva* genome has an overall stable gene complement but harbours many long chromosomal inversions that were more frequent in isolates from its native range. Furthermore, putative lineage-specific expansion of TEs increased the level of genome compartmentalization. Finally, the genomes provided herein will serve as an important resource for future comparative genomic analyses among members of the Dothideomycetes. In particular, association and transcriptomic studies can provide further insights into the phenotypic consequences and transcriptional impact of long inversions in *S. musiva*. Moreover, future studies can shed light on possible associations between TE expansion or the presence/absence of effector genes and adaptation to *Populus* spp.

## Supplementary material

10.1099/mgen.0.001603 Uncited Supplementary Material 1.

10.1099/mgen.0.001603 Uncited Supplementary Material 2.

## Abbreviations

ABC: ATP-binding cassette
CAZyme: carbohydrate active enzyme
Chr: chromosome
GO: Gene Ontology
HOG: hierarchical orthologous group
LTR: long terminal repeat
MFS: major facilitator superfamily
NCBI: National Center for Biotechnology Information
NRPS: nonribosomal peptide synthetase
ONT: Oxford Nanopore Technology
PKS: polyketide synthase
PL: polysaccharide lyase
RIP: repeat-induced point
SRA: Sequence Read Archive
TE: transposable element
