# Supplementary material for: The poplar pathogen Sphaerulina musiva has a dynamic genome architecture marked by chromosomal inversions and changes in transposable element abundance
Source: Microb Genom. 2026 Jan 7;12(1):001603. doi: 10.1099/mgen.0.001603 (PMC12778737; doi:10.1099/mgen.0.001603)
Supplement: Uncited Supplementary Material 1. [file mgen-12-01603-s001.pdf]

The poplar pathogen *Sphaerulina musiva* has a dynamic genome architecture marked by chromosomal inversions and changes in transposable element abundance

Alex Z. Zaccaron[1], Alexandre Lassagne[1], Kelsey L. Søndreli[1], Martha A. Sudermann[1], Ricardo I. Alcalá Briseño[1,*], Niklaus J. Grünwald[3], Alexandra J. Weisberg[1], Jared M. LeBoldus[1,2,#]

[1] Department of Botany and Plant Pathology, Oregon State University, Corvallis, OR, USA.

[2] Department of Forest Engineering, Resources, and Management, Oregon State University, Corvallis, OR, USA

[3] Horticultural Crops Disease and Pest Management Research Unit, USDA Agricultural Research Service, Corvallis, OR, USA

* Current address: Plant Pathology and Environmental Microbiology, Pennsylvania State University, University Park, PA.

Correspondence: Jared M. LeBoldus. Department of Botany and Plant Pathology, Oregon State University, 2701 SW Campus Way, 2503 Cordley Hall, Corvallis, OR 97331, USA; Department of Forest Engineering, Resources, and Management, Oregon State University, 140 Peavy Forest Science Center, 3100 SW Jefferson Way, Corvallis, OR 97331, USA. E-mail address: Jared.LeBoldus@oregonstate.edu. Alexandra J. Weisberg. Department of Botany and Plant Pathology, Oregon State University, 2701 SW Campus Way, 2503 Cordley Hall, Corvallis, OR 97331, USA. E-mail address: Alexandra.Weisberg@oregonstate.edu.

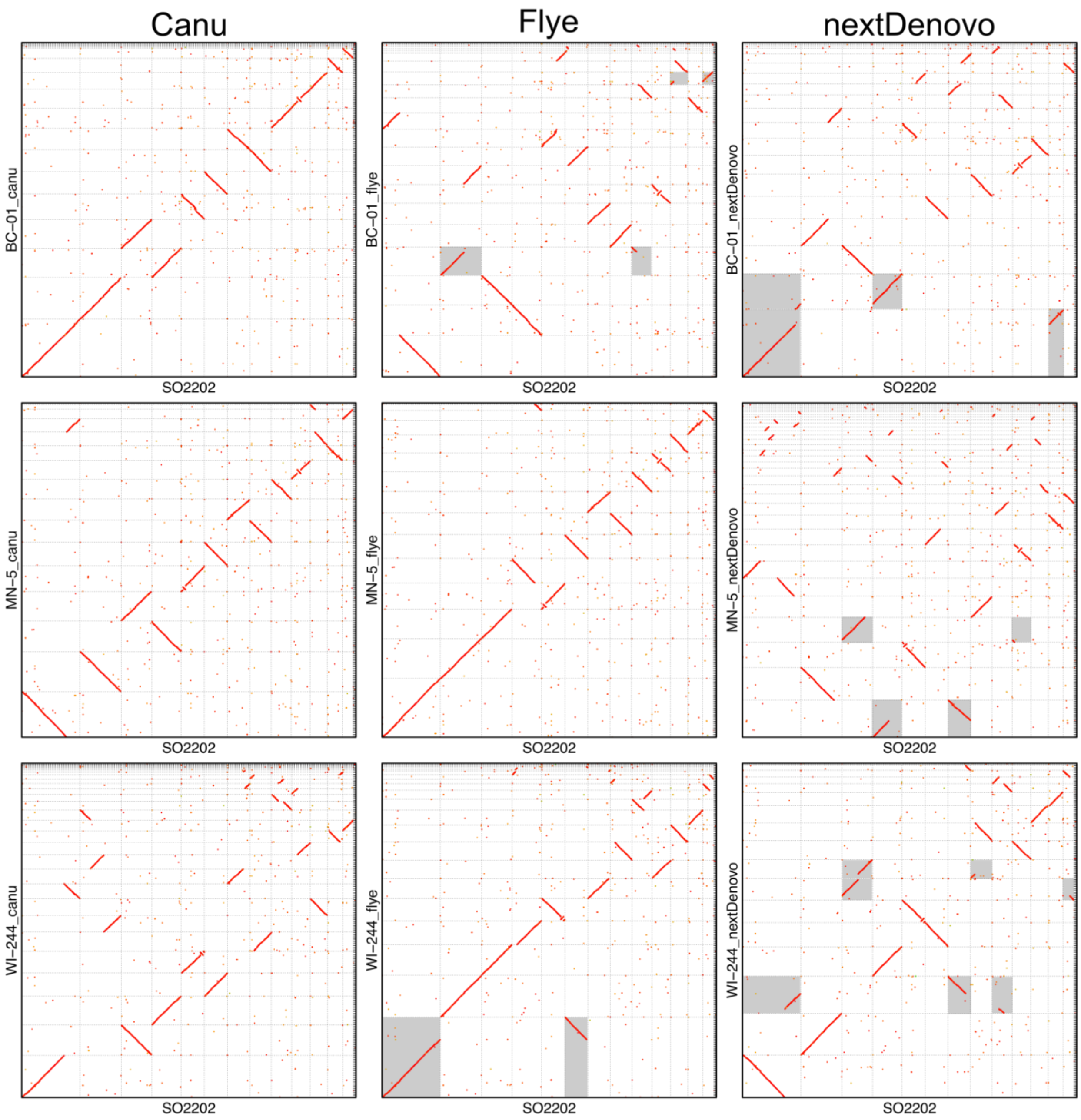

Fig. S1. Examples of chimeric contigs assembled by Flye and nextDenovo. The figure shows whole-genome alignment dotplots of assemblies generated with Canu, Flye, and nextDenovo for *Sphaerulina musiva* isolates BC-01, MN-5, and WI-244 compared to the reference genome assembly of isolate SO2202 (GCA_000320565.2). Chimeric alignments are highlighted. These chimeric alignments reveal assembled contigs that map to different scaffolds of the reference genome of isolate SO2202. The figure shows that Flye produced two and one chimeric contigs for isolates BC-01 and WI-244, respectively, and that nextDenovo produced two, two, and three chimeric contigs for isolates BC-01, MN-5, and WI-244, respectively. Dashed lines delineate contigs boundaries. Contigs were sorted by size.

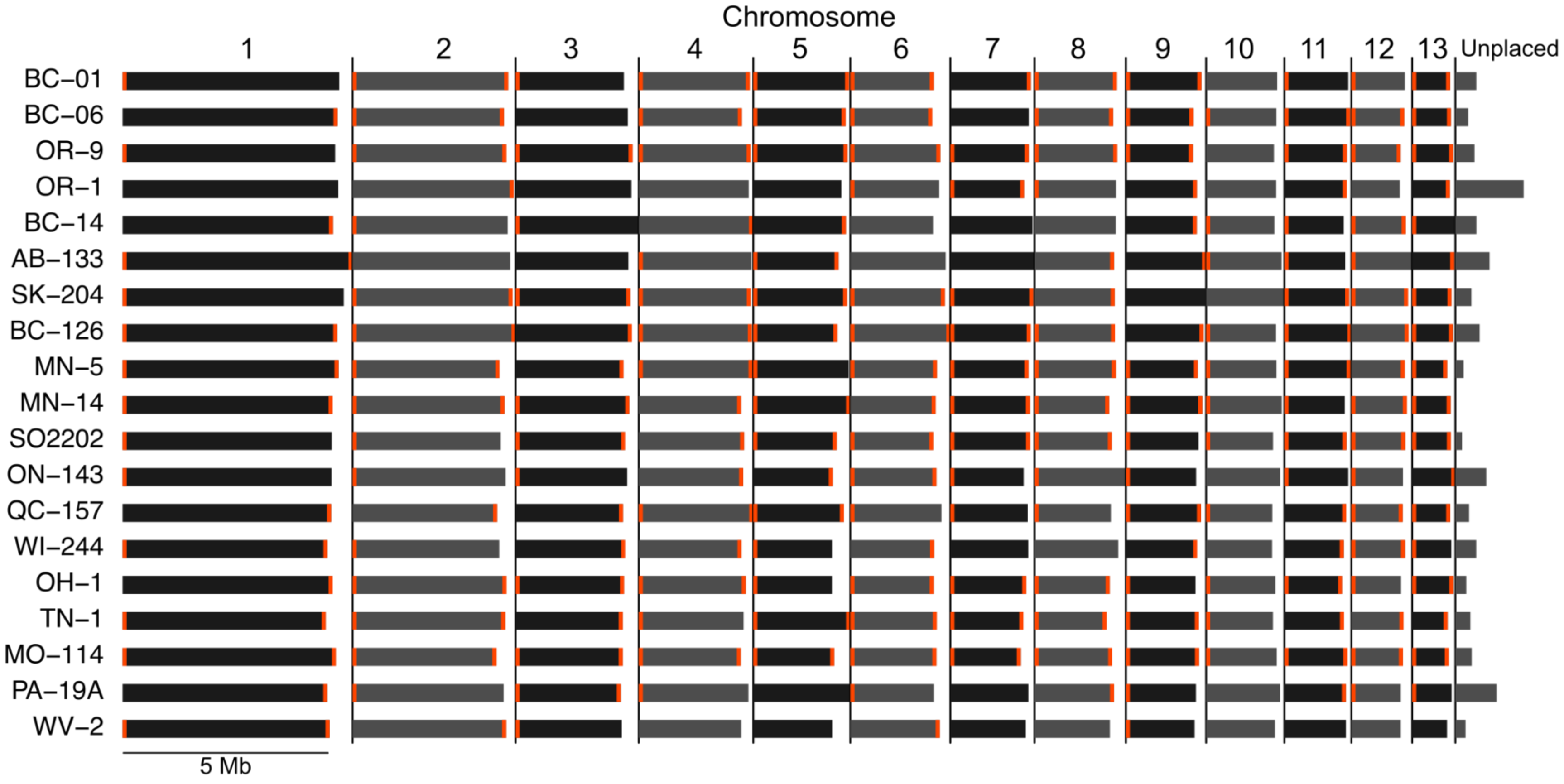

Fig. S2. Comparison of the size of the assembled chromosomes of *Sphaerulina musiva* isolates. The figure shows the size in scale of the 13 assembled chromosomes. Unplaced corresponds to the total size of unplaced contigs. Chromosomes containing telomeric repeats (GGGTTAx3 or TAACCCx3) at the first or last 300 bp are indicated with red rectangles covering the first or last 100 kb of the chromosomes.

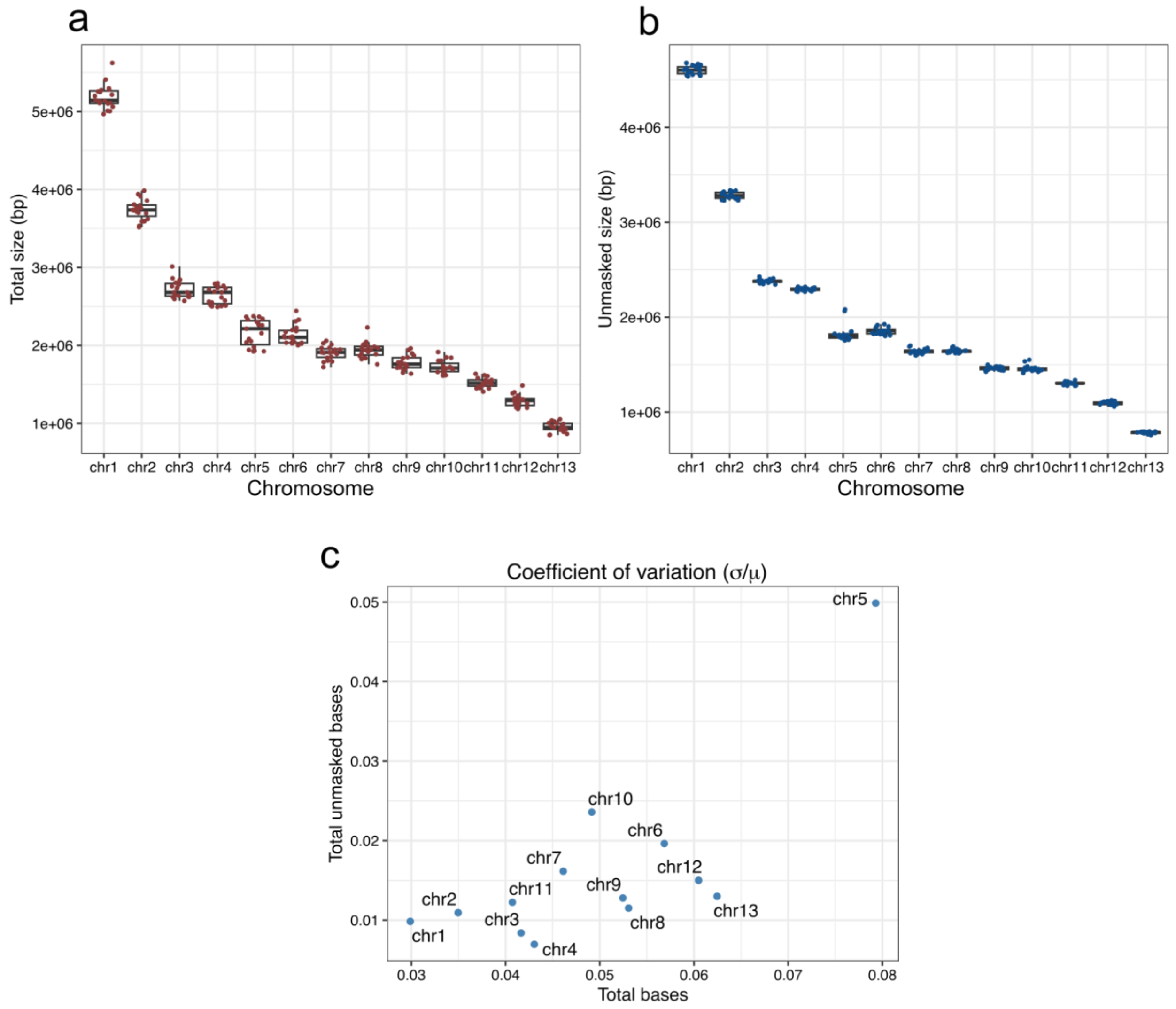

Fig. S3. Difference in size of chromosomes from different isolates of *Sphaerulina musiva* is explained by the presence of repetitive DNA. (a) Box plots show variation of the size of the chromosomes of 19 *S. musiva* isolates when considering all assembled base pairs. (b) Similar to (a), but instead of all base pairs, only base pairs not masked after repetitive DNA masking were considered. (c) Scatter plot showing the coefficient of variation, i.e., standard deviation divided by the mean, of the total size of the chromosomes among the 19 isolates (x-axis) and the total size of unmasked bases (y-axis).

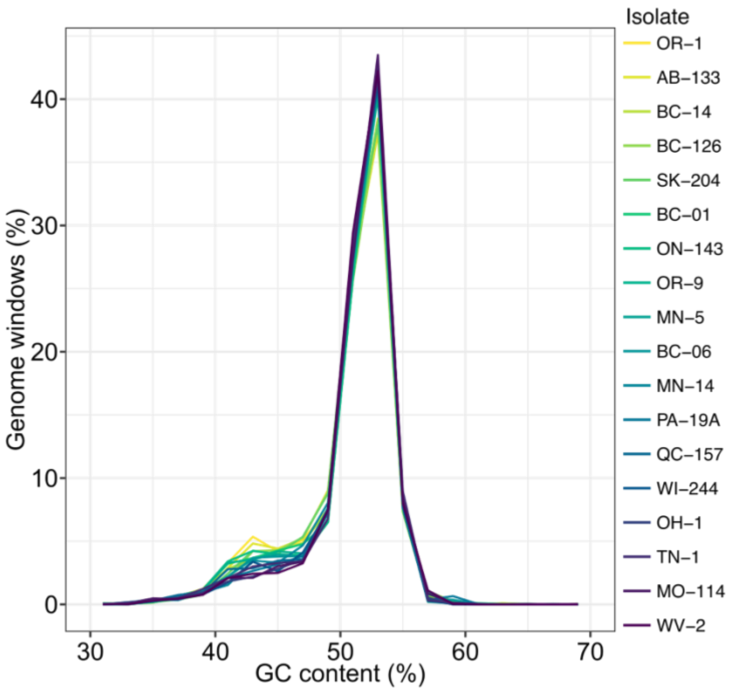

Fig. S4. *Sphaerulina musiva* exhibits bimodal GC distribution. Distribution of GC content of the genome assemblies based on a 10 kb sliding window. Lines representing genomes were color-coded based on their repetitive DNA content, from lowest in dark purple to highest in yellow. A second peak at approximately 43% GC is more visible for genomes with high repeat content.

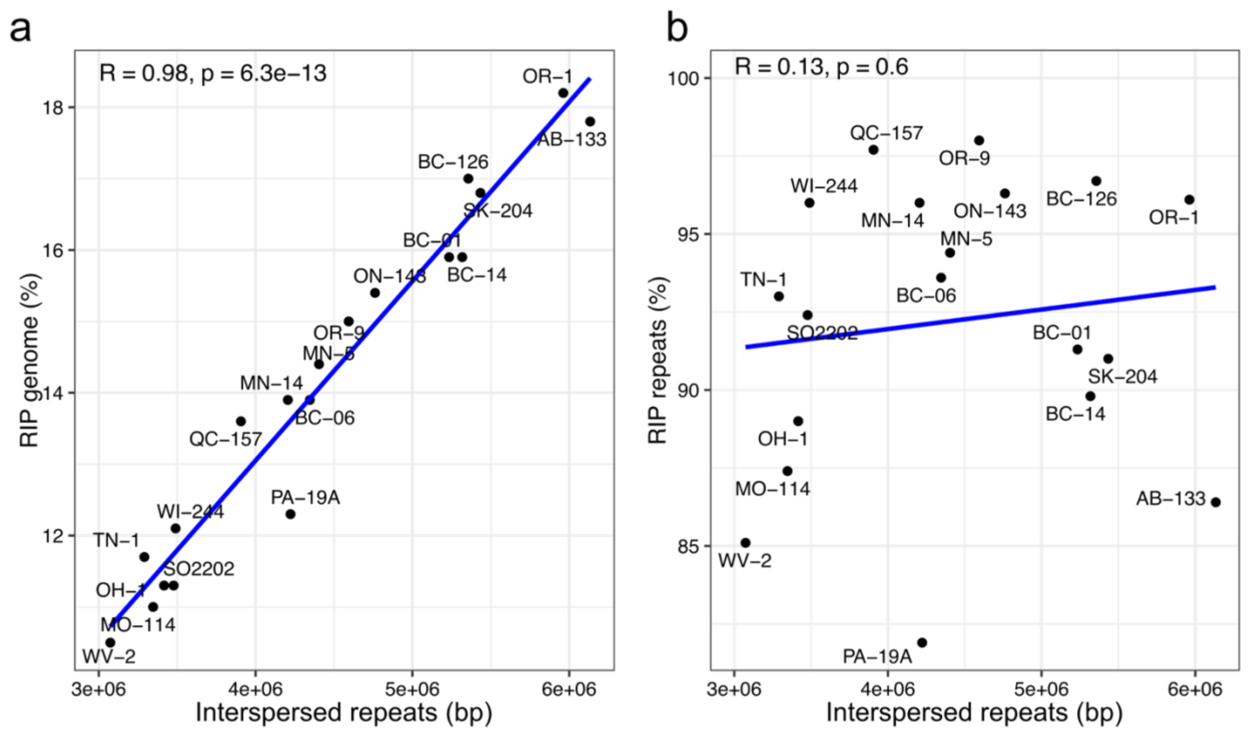

Fig. S5. The genome of *Sphaerulina musiva* shows no correlation between abundance of repetitive DNA and percentage of repeats affected by Repeat-Induced Point mutations. (a) Scatter plot showing positive correlation between abundance of repetitive DNA and percentage of the genomes affected by RIP. (b) Scatter plot showing poor correlation between abundance of repetitive DNA and percentage of repeats affected by RIP. Blue lines represent simple line regression on the values of X and Y axes. Pearson correlation coefficients and p-values are shown at the top of each plot.

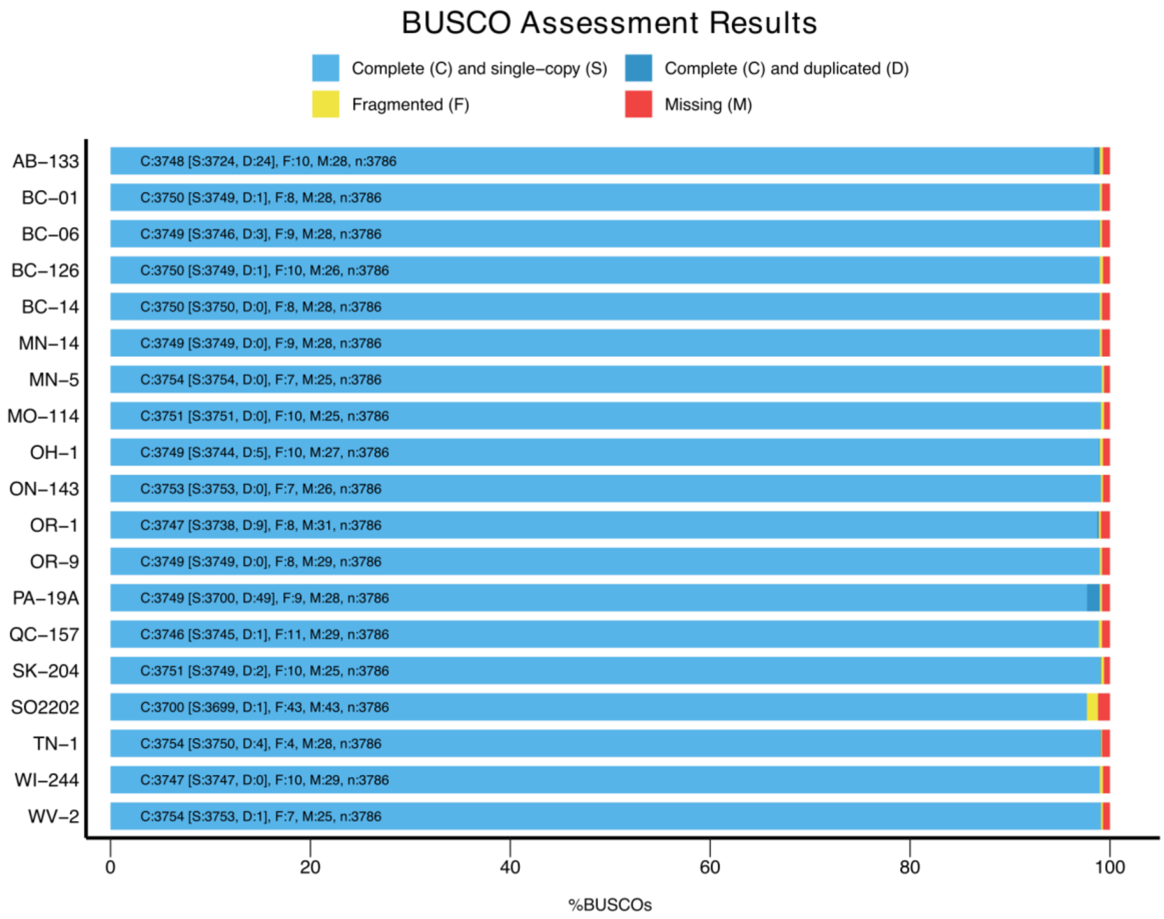

Fig. S6. Comparison of BUSCO completeness of *Sphaerulina musiva* isolates. Values were obtained with BUSCO v5.7.1 in protein mode, using the Dothideomycetes_odb10 (2024-01-08) data base containing 3,786 BUSCOs as reference. For comparison, values for the previous reference genome of *S. musiva* S02202 (GCA_000320565.2) are also shown.

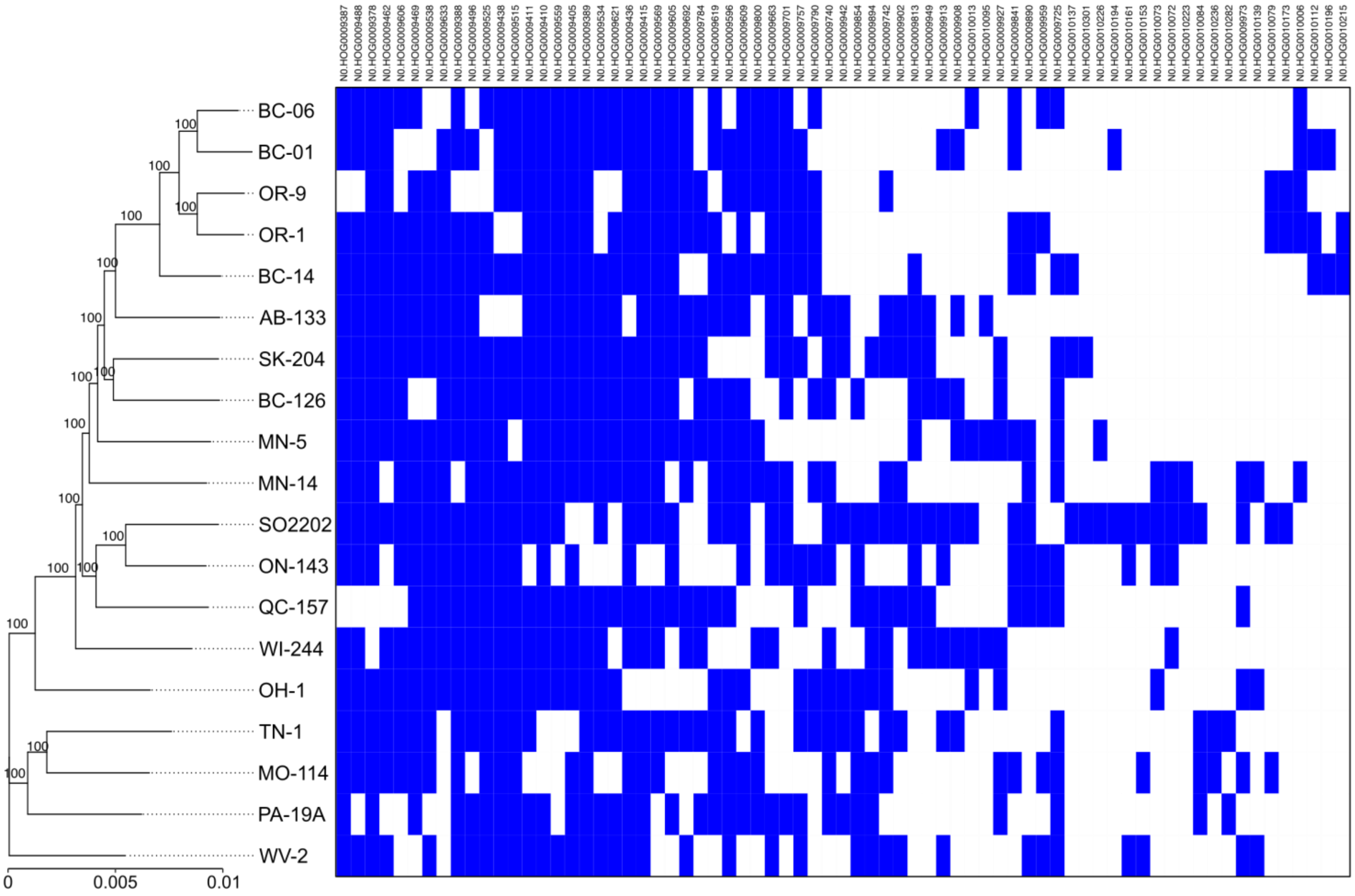

Fig. S7. Presence/absence variation of accessory hierarchical orthogroups (HOGs) containing candidate effector genes from isolates of *Sphaerulina musiva*. The figure shows a maximum likelihood phylogenetic tree constructed based on coding sequences of 1,620 complete single-copy BUSCO genes. Values above branches indicate bootstrap support based on 1000 ultrafast bootstrap replicates. The heatmap shows presence (blue) and absence (white) of genes in HOGs containing candidate effectors.

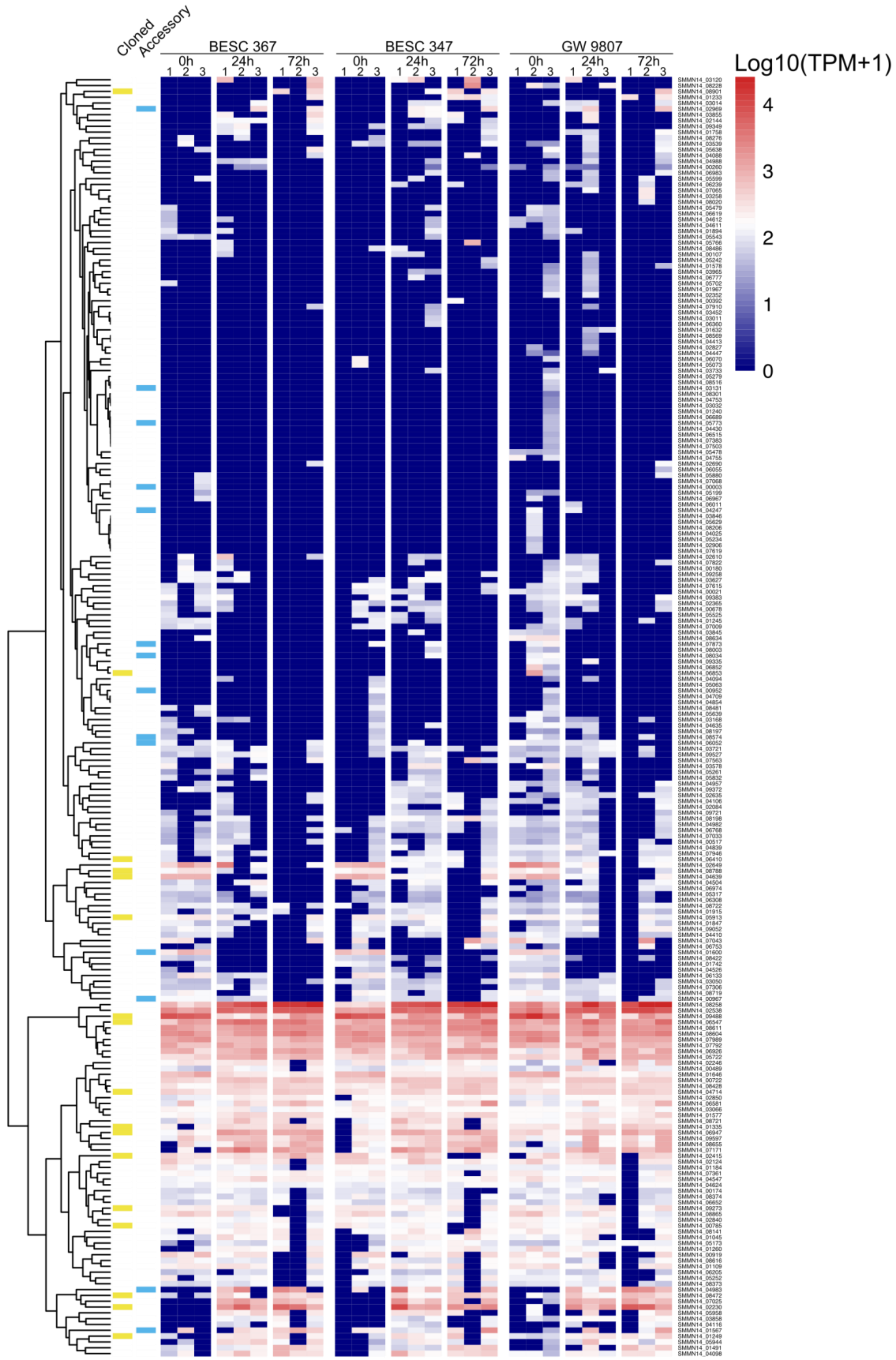
Cloned
Accessory
BESC 367
BESC 347
GW 9807
0h
24h
72h
1 2 3
Log10(TPM+1)
4
3
2
1
0

Fig. S8. Expression of candidate effector genes in *Sphaerulina musiva* isolate MN-14 during interaction with *Populus trichocarpa*. Expression is shown for three time points, i.e., 0, 24, and 27 h after inoculation during interaction with *P. trichocarpa* genotypes BESC367, BESC347, and GW9807. Genes considered accessory, i.e., not predicted in all *S. musiva* isolates, are indicated on the left-hand side. Candidate effectors previously cloned and studied by Zhao et al (2023) (doi:10.1094/MPMI-07-23-0091-R) are also indicated. Candidate effector genes for which expression was considered zero in all conditions were omitted. Expression is shown in logarithmic scale (base 10) of transcripts per million (TPM) + 1. Genes with low raw read counts (< 10) received TPM equal to 0. TPM values for all candidate effector genes are shown in Table S9. Genes for which the TPM values were considered 0 in all samples were omitted.

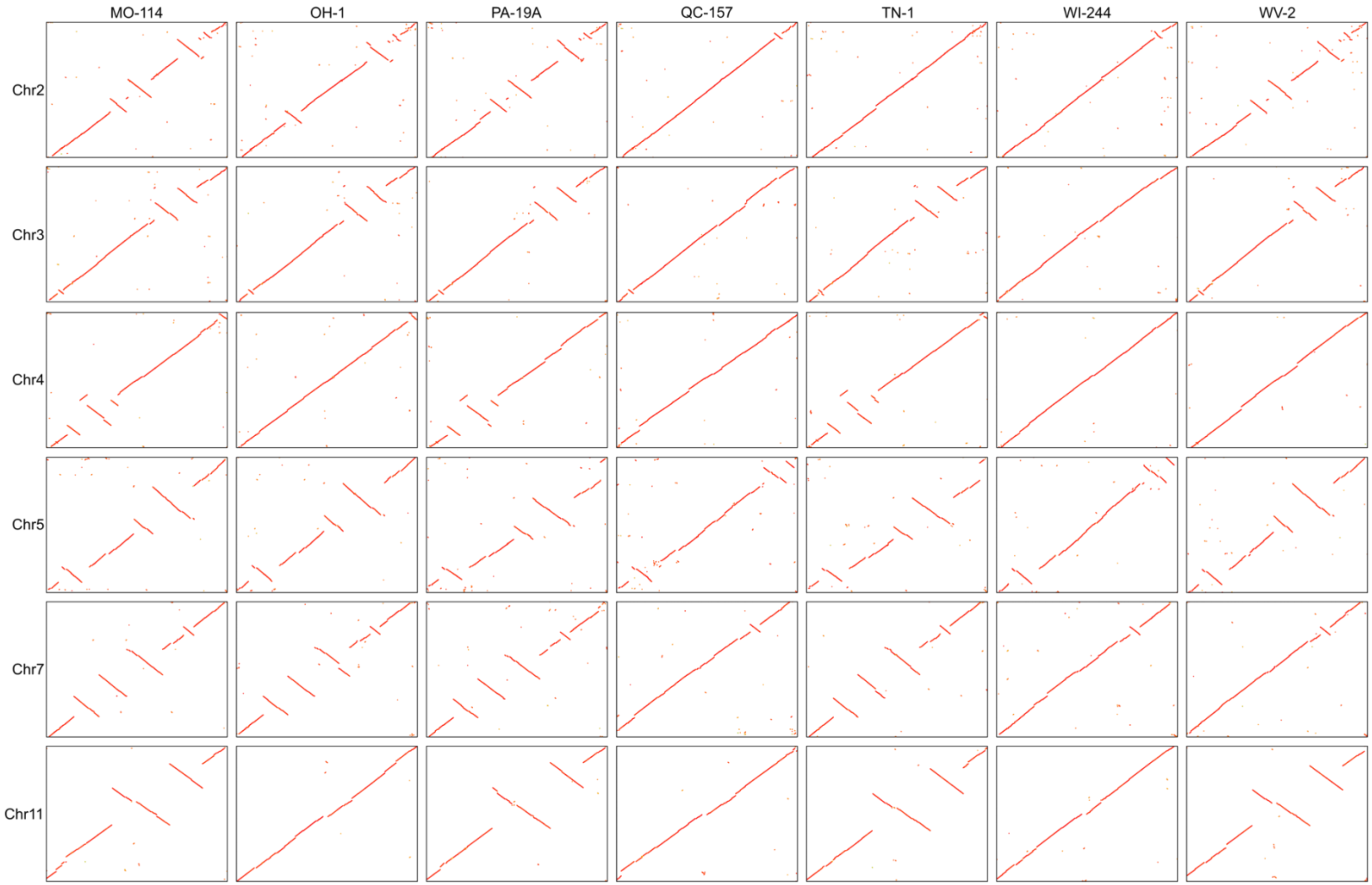

Fig. S9. Presence of tandem or proximal long chromosomal inversions in the genomes of *Sphaerulina musiva* isolates. The figure shows alignment dotplots of five pseudochromosomes in six *S. musiva* isolates against the reference pseudochromosomes of isolate MN-14. The dotplots were generated based on whole pseudochromosome alignments at the nucleotide level. In each plot, the reference sequence (MN-14) is shown on the x-axis, and sequences from other isolates on the y-axis

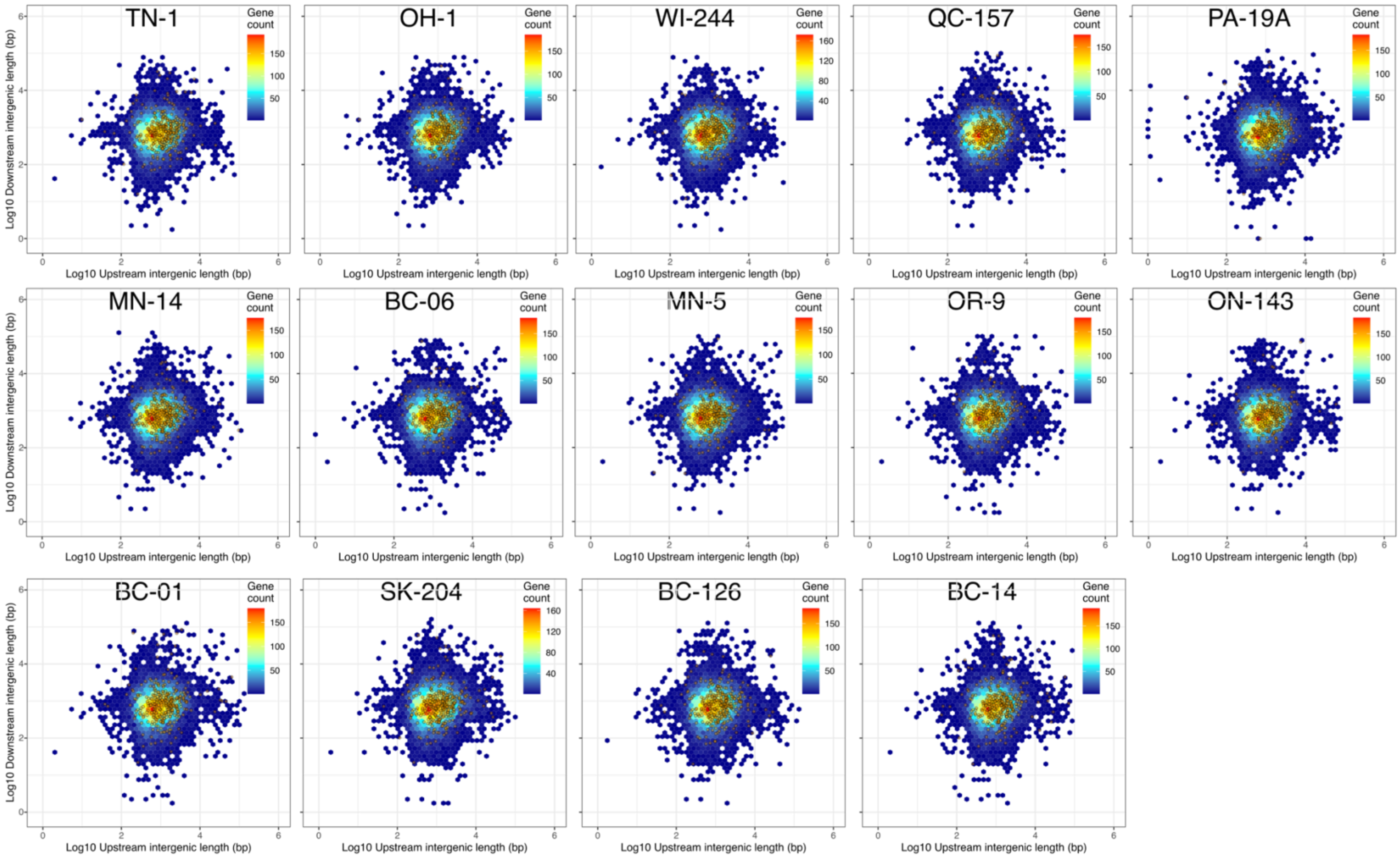

Fig. S10. Density plots of intergenic regions of all predicted genes for 16 isolates of *Sphaerulina musiva*. Isolate names are shown at the top of each plot. Plots were organized from top left to bottom right based on the percentage of interspersed repeat content of the genomes. Candidate effectors are shown as transparent points. Plots for isolates WV-2, MO-114, AB-133, and OR-1 are shown in Fig. 4 of the main manuscript
